# Supplementary material for: The Arabidopsis thaliana mobilome and its impact at the species level
Source: eLife. 2016 Jun 3;5:e15716. doi: 10.7554/eLife.15716 (PMC4917339; doi:10.7554/eLife.15716)
Supplement: Figure 1—source data 3. — DOI: http://dx.doi.org/10.7554/eLife.15716.005 [file elife-15716-fig1-data3.zip › Figure1Source_data3.pdf]

Figure 1 - Source data 3

Family	# Insertions
**COPIA**	**1408**
ATCOPIA78	279
META1	172
ATCOPIA93	132
ATCOPIA28	86
ATCOPIA4	75
ATCOPIA21	53
ATCOPIA52	43
ATCOPIA31	40
ATCOPIA36	39
ATCOPIA23	36
ATCOPIA3	35
ATCOPIA57	35
ATCOPIA63	35
ATCOPIA72	33
ENDOVIR1	32
ATCOPIA2	31
ATCOPIA69	28
ATCOPIA12	26
ATCOPIA49	20
ATCOPIA75	19
ATCOPIA74	15
ATCOPIA11	13
ATRE1	13
ATCOPIA26	10
ATCOPIA27	10
ATCOPIA35	9
ATCOPIA22	8
ATCOPIA45	8
ATCOPIA48	8
ATCOPIA85	8
ATCOPIA13	7
ATCOPIA65	7
ATCOPIA58	6
ATCOPIA56	4
ATCOPIA90	4
ATCOPIA15	3
ATCOPIA62	3
ATCOPIA16	2
ATCOPIA67	2
ATCOPIA89	2
ATCOPIA8B	2
ATCOPIA97	2
ATCOPIA1	1
ATCOPIA10	1
ATCOPIA14	1
ATCOPIA18	1
ATCOPIA20	1
ATCOPIA25	1
ATCOPIA32	1
ATCOPIA47	1
ATCOPIA55	1
ATCOPIA79	1
ATCOPIA8A	1
ATCOPIA95	1
TA1-AT	1
**Gypsy**	**34**
ATGP1	13
ATGP3	8
ATLANTYS2	5
ATGP9B	4
ATGP2	2
ATLANTYS1	1
TA1-2	1
**LINE**	**85**
ATLINEIII	41
ATLINE2	21
ATLINE1A	12
ATLINE1_1	5
ATLINE1_2	3
ATLINE1_5	2
TSCL	1

**MuDR**	**729**
VANDAL6	95
ATDNA2T9A	83
VANDAL14	47
VANDAL17	44
AT9NMU1	41
ATMU4	39
VANDAL2N1	34
VANDAL1	33
VANDAL3	29
VANDAL21	28
VANDAL22	26
VANDAL16	23
VANDAL5	20
ATDNAI26T9	16
ARNOLD2	14
ATMU6	14
ATDNA2T9B	13
ATMU3	11
VANDAL1N1	11
ATDNA2T9C	10
ATDNAI27T9B	10
ATMU1	10
ATMU7	10
AT9TSD1	9
ATDNA1T9A	9
ARNOLDY2	8
ATMU3N1	8
ATMU6N1	6
BOMZH1	5
AT9MU1	4
ATDNAI27T9C	4
ATMU2	3
VANDAL20	3
ATMU10	2
VANDAL13	2
ARNOLD1	1
ARNOLDY1	1
BRODYAGA1	1
BRODYAGA1A	1
VANDAL11	1
**En-Spm**	**185**
ATENSPM5	55
ATENSPM9	50
ATENSPM2	25
ATENSPM3	18
ATENSPM1A	15
ATENSPM4	13
ATENSPM1	8
ATENSPM11	1
**hAT**	**303**
TAG1	126
SIMPLEHAT2	69
ATHAT3	37
TAG2	32
SIMPLEHAT1	23
ATHATN6	13
ATHAT7	2
ATHATN10	1
**Pogo**	**7**
ATHPOGON3	7
**Harbinger**	**70**
HARBINGER	48
SIMPLEGUY1	10
**DNA**	**14**
ATDNA12T3A	6
ATTIR16T3A	8
