# Supplementary material for: The Arabidopsis thaliana mobilome and its impact at the species level
Source: eLife. 2016 Jun 3;5:e15716. doi: 10.7554/eLife.15716 (PMC4917339; doi:10.7554/eLife.15716)
Supplement: Supplementary file 1. — Distribution of CN values, Manhattan plot and QQ-plot across the joined data set (391 accessions) for the indicated TE families. Summary statistics of associations are indicated below. MAF indicates Minor Allele Frequency in the joined dataset. Genes within GWAS intervals are indicated (MET2a is in bold). DOI: http://dx.doi.org/10.7554/eLife.15716.023 [file elife-15716-supp1.zip › Supplementary_file1.pdf]

Supplementary file 1. GWAS results for CNV

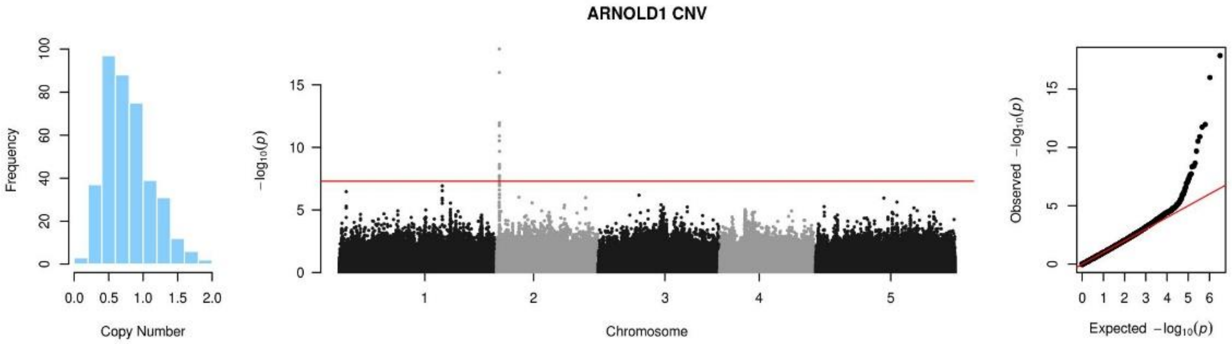

TE	GWAS associated interval	Leading SNP	Type of association	*P-value* Joined	Worldwide	Sweden	MAF	Candidate gene(s)
ARNOLD1	Chr2:550102-595539	Chr2-560680	*CIS*	1.39E-18	2.51E-09	1.69E-08	0.077	

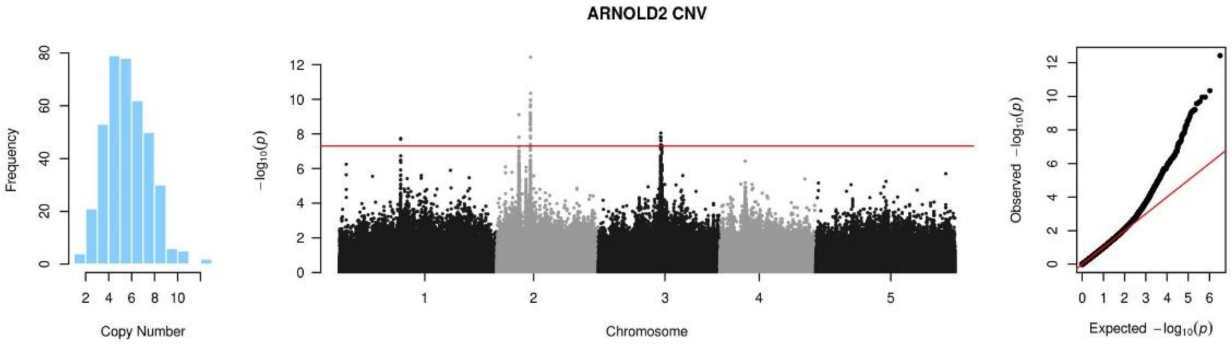

TE	GWAS associated interval	Leading SNP	Type of association	*P-value* Joined	Worldwide	Sweden	MAF	Candidate gene(s)
ARNOLD2	Chr1:11886940-11900534	Chr1-11886941	*CIS*	2.02E-08	6.12E-05	2.78E-05	0.218	
ARNOLD2	Chr2:4360249-4361088	Chr2-4360250	*TRANS*	7.77E-10	3.68E-07	3.57E-05	0.269	*AT2G11010, AT2G11015*
ARNOLD2	Chr2:6531910-6609313	Chr2-6557527	*CIS*	1.66E-09	4.30E-03	5.10E-05	0.150	
ARNOLD2	Chr3:12047135-12123371	Chr3-12098457	*TRANS*	8.96E-09	9.98E-06	1.04E-04	0.474	*AT3G30430, AT3G30456, AT3G30460, AT3G30520, AT3G30530*

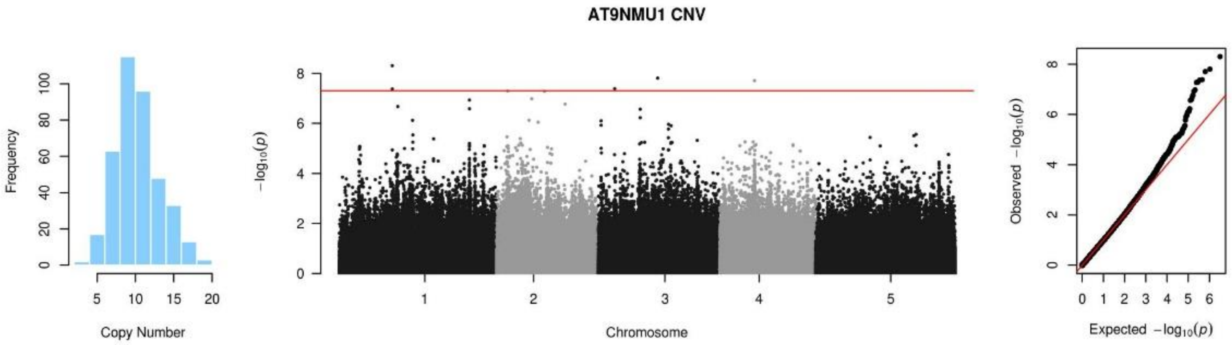

TE	GWAS associated interval	Leading SNP	Type of association	*P-value* Joined	Worldwide	Sweden	MAF	Candidate gene(s)
AT9NMU1	Chr1:10280547-10280549	Chr1-10280548	*TRANS*	4.97E-09	2.99E-07	NA	0.344	*AT1G29350, AT1G29355, AT1G29357, AT1G29370, AT1G29380, AT1G29390, AT1G29395, AT1G29400*
AT9NMU1	Chr3:11495299-11495301	Chr3-11495300	*TRANS*	1.56E-08	1.85E-08	NA	0.228	*AT3G29644*
AT9NMU1	Chr4:6766764-6766766	Chr4-6766765	*TRANS*	1.97E-08	3.96E-05	1.24E-04	0.146	*AT4G11070*

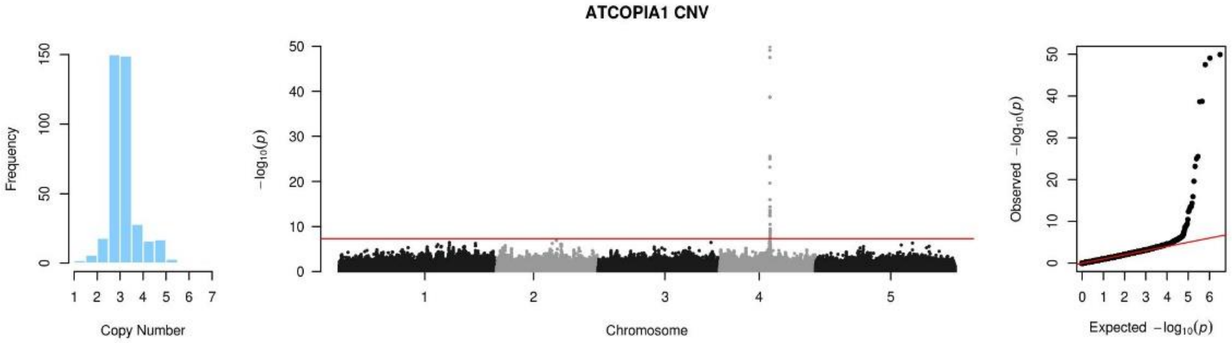

TE	GWAS associated interval	Leading SNP	Type of association	*P-value* Joined	Worldwide	Sweden	MAF	Candidate gene(s)
ATCOPIA1	Chr4:9712617-9782935	Chr4-9733104	*CIS*	7.70E-50	2.24E-24	8.83E-29	0.095	

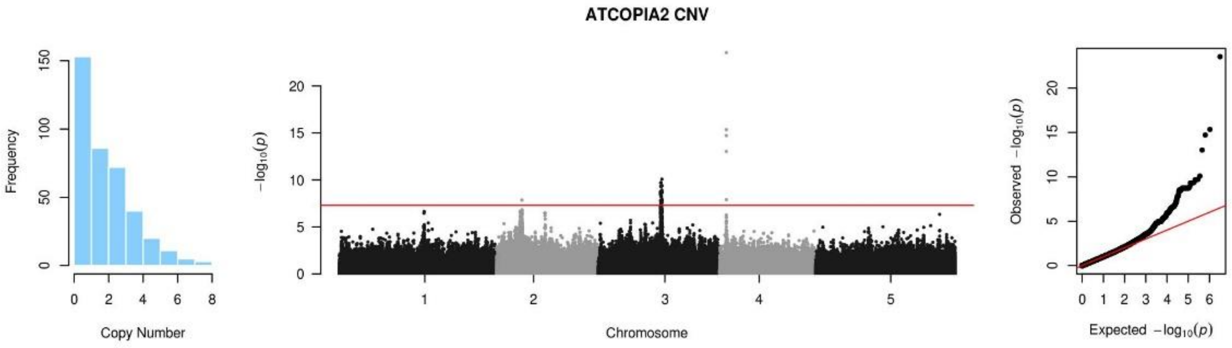

TE	GWAS associated interval	Leading SNP	Type of association	*P-value* Joined	Worldwide	Sweden	MAF	Candidate gene(s)
ATCOPIA2	Chr2:4934115-4934117	Chr2-4934116	*TRANS*	1.37E-08	1.66E-03	2.49E-08	0.254	*AT2G12290*
ATCOPIA2	Chr3:12010819-12354238	Chr3-12315559	*CIS*	8.18E-11	7.97E-09	NA	0.118	
ATCOPIA2	Chr4:1313271-1317155	Chr4-1316584	*CIS*	2.86E-24	2.51E-11	3.49E-18	0.485	

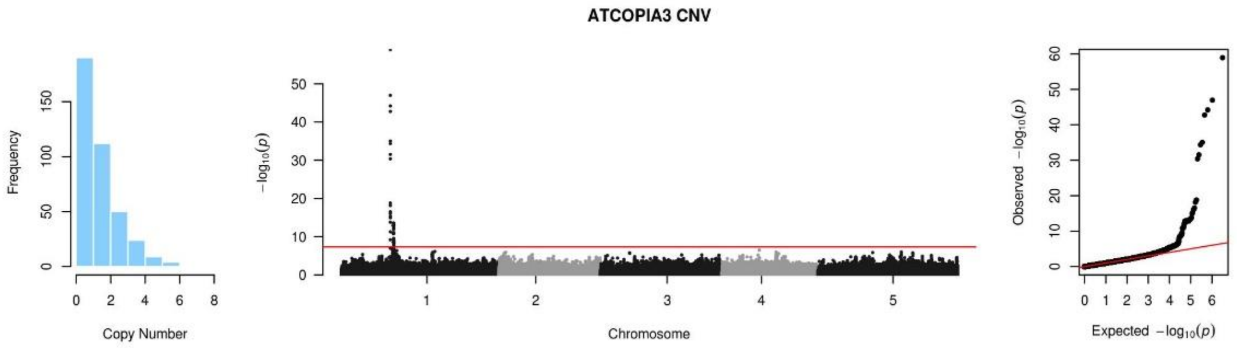

TE	GWAS associated interval	Leading SNP	Type of association	*P-value* Joined	Worldwide	Sweden	MAF	Candidate gene(s)
ATCOPIA3	Chr1:9476030-9480581	Chr1-9478025	*CIS*	1.18E-59	4.27E-23	4.48E-41	0.479	
ATCOPIA3	Chr1:10065788-10144540	Chr1-10114390	*TRANS*	2.77E-10	1.00E-03	8.50E-08	0.244	Centromere

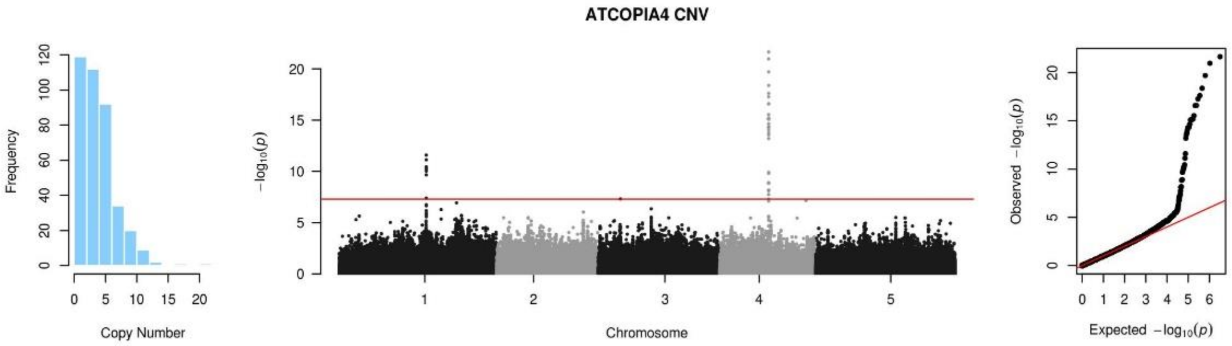

TE	GWAS associated interval	Leading SNP	Type of association	*P-value*			MAF	Candidate gene(s)
				Joined	Worldwide	Sweden		
ATCOPIA4	Chr1:16853511-16857178	Chr1-16853512	*CIS*	7.37E-12	1.82E-08	6.36E-05	0.456	
ATCOPIA4	Chr4:9483993-9488516	Chr4-9485781	*CIS*	2.24E-22	8.60E-14	7.06E-11	0.477	

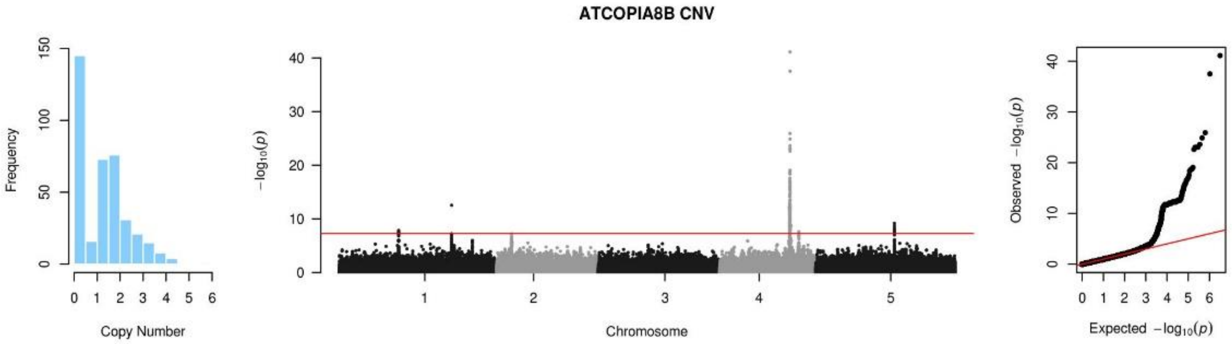

TE	GWAS associated interval	Leading SNP	Type of association	*P-value*			MAF	Candidate gene(s)
				Joined	Worldwide	Sweden		
ATCOPIA8B	Chr1:11505352-11506731	Chr1-11505353	*TRANS*	1.44E-08	1.09E-04	1.65E-05	0.300	*AT1G32000, AT1G32010, AT1G32020, AT1G32030*
ATCOPIA8B	Chr1:21752025-21752027	Chr1-21752026	*CIS*	2.90E-13	2.17E-05	1.42E-10	0.238	
ATCOPIA8B	Chr4:13531245-13816484	Chr4-13627337	*CIS*	7.46E-42	5.10E-28	8.82E-17	0.454	
ATCOPIA8B	Chr4:15327376-15327920	Chr4-15327377	*CIS*	2.63E-08	NA	1.10E-07	0.131	
ATCOPIA8B	Chr5:15241272-15244051	Chr5-15241273	*TRANS*	6.73E-10	2.96E-04	1.28E-07	0.413	*AT5G38195*

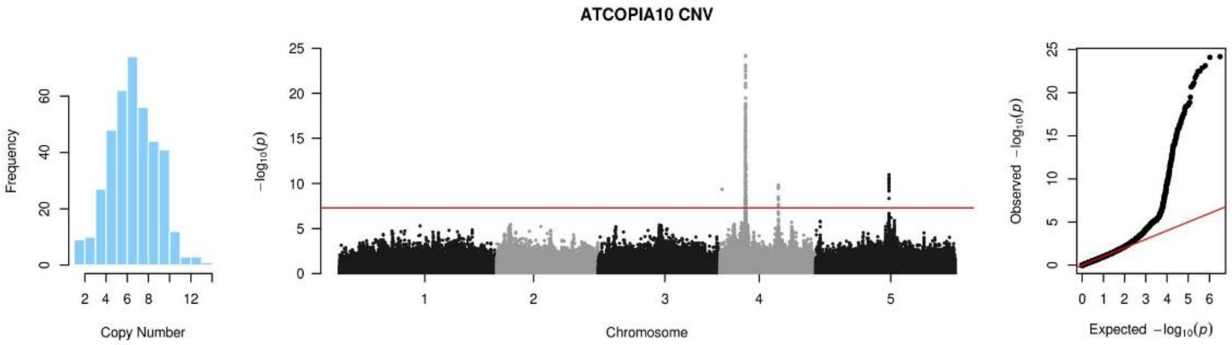

TE	GWAS associated interval	Leading SNP	Type of association	*P-value* Joined	Worldwide	Sweden	MAF	Candidate gene(s)
ATCOPIA10	Chr4:500303-500305	Chr4-500304	*TRANS*	4.49E-10	1.34E-05	6.75E-06	0.269	*AT4G01140, AT4G01150, AT4G01160, AT4G01170, AT4G01180, AT4G01190, AT4G01200, AT4G01210*
ATCOPIA10	Chr4:4907701-5122395	Chr4-5034442	*CIS*	7.99E-25	9.13E-16	2.21E-10	0.297	
ATCOPIA10	Chr4:11364113-11380407	Chr4-11376940	*CIS*	1.54E-10	2.75E-07	7.48E-08	0.179	
ATCOPIA10	Chr5:14196459-14209447	Chr5-14196547	*CIS*	1.04E-11	4.65E-07	6.95E-07	0.362	

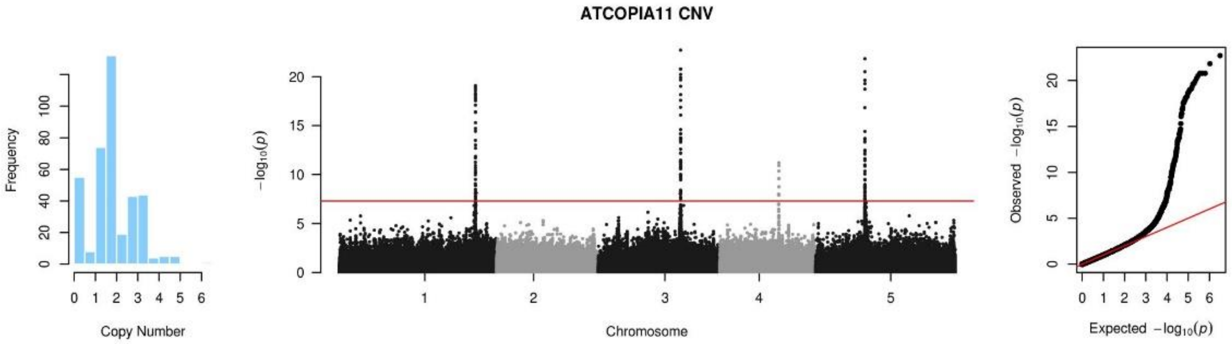

TE	GWAS associated interval	Leading SNP	Type of association	*P-value* Joined	Worldwide	Sweden	MAF	Candidate gene(s)
ATCOPIA11	Chr1:26341260-26465474	Chr1-26370884	*CIS*	8.42E-20	1.06E-08	2.03E-13	0.300	
ATCOPIA11	Chr3:15867269-15931805	Chr3-15917909	*CIS*	1.95E-23	5.97E-16	1.08E-08	0.164	
ATCOPIA11	Chr4:11441725-11484545	Chr4-11460817	*TRANS*	1.16E-08	NA	5.65E-06	0.078	*AT4G21500, AT4G21510, AT4G21520, AT4G21530, AT4G21534, AT4G21540, AT4G21550, AT4G21560, AT4G21570, AT4G21580, AT4G21585, AT4G21590*
ATCOPIA11	Chr5:9529127-9560997	Chr5-9540237	*CIS*	1.48E-22	3.20E-13	1.04E-10	0.249	

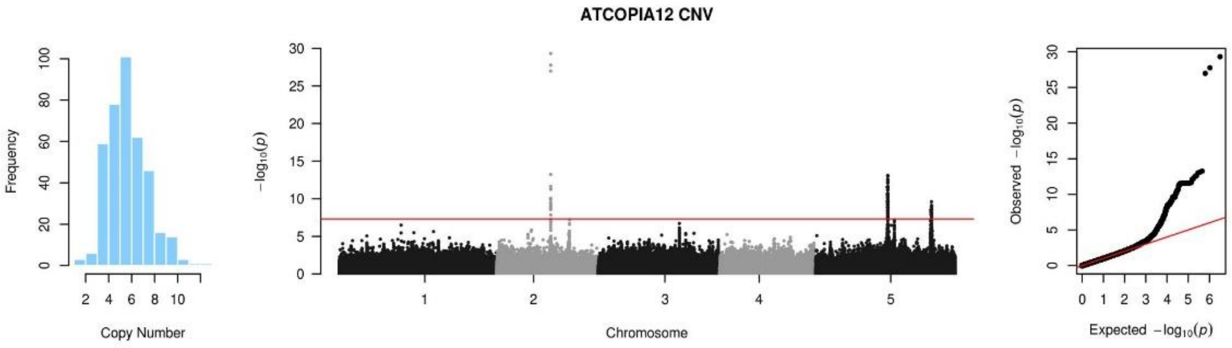

TE	GWAS associated interval	Leading SNP	Type of association	*P-value*			MAF	Candidate gene(s)
				Joined	Worldwide	Sweden		
ATCOPIA12	Chr2:10485359-10493333	Chr2-10490467	*CIS*	4.84E-30	1.14E-12	3.35E-19	0.415	
ATCOPIA12	Chr5:13941203-13960559	Chr5-13948475	*CIS*	9.90E-14	1.37E-08	1.06E-06	0.303	
ATCOPIA12	Chr5:22334744-22450916	Chr5-22388168	*CIS*	2.53E-10	7.56E-05	8.42E-08	0.079	

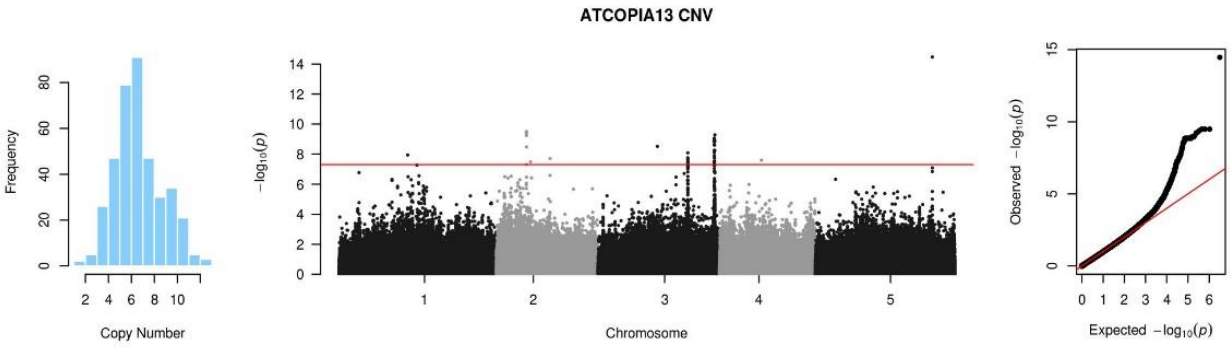

TE	GWAS associated interval	Leading SNP	Type of association	*P-value*			MAF	Candidate gene(s)
				Joined	Worldwide	Sweden		
ATCOPIA13	Chr2:10431596-10431598	Chr2-10431597	*TRANS*	1.97E-08	1.41E-05	4.39E-03	0.423	*AT2G24570*
ATCOPIA13	Chr2:5853595-5856494	Chr2-5853596	*CIS*	4.00E-10	8.09E-08	5.63E-04	0.138	
ATCOPIA13	Chr3:11495299-11495301	Chr3-11495300	*TRANS*	3.02E-09	4.13E-09	NA	0.228	*AT3G29644*
ATCOPIA13	Chr3:17353731-17359266	Chr3-17356687	*TRANS*	8.16E-09	1.60E-04	6.65E-06	0.221	*AT3G47090, AT3G47100, AT3G47110, AT3G47120, AT3G47130, AT3G47140, AT3G47150, AT3G47160, AT3G47170*
ATCOPIA13	Chr3:22502613-22593700	Chr3-22593699	*TRANS*	5.18E-10	3.94E-04	8.16E-08	0.274	*AT3G60870, AT3G60880, AT3G60890, AT3G60897, AT3G60900, AT3G60910, AT3G60920, AT3G60940, AT3G60950, AT3G60960, AT3G60961, AT3G60966, AT3G60970, AT3G60972, AT3G60980, AT3G60990, AT3G61010, AT3G61028, AT3G61030, AT3G61035, AT3G61040, AT3G61050, AT3G61060*
ATCOPIA13	Chr4:8148311-8148313	Chr4-8148312	*TRANS*	2.52E-08	2.38E-07	NA	0.226	***AT4G14140 (MET2a)***
ATCOPIA13	Chr5:22617655-22617657	Chr5-22617656	*CIS*	3.38E-15	4.29E-08	6.58E-09	0.151	

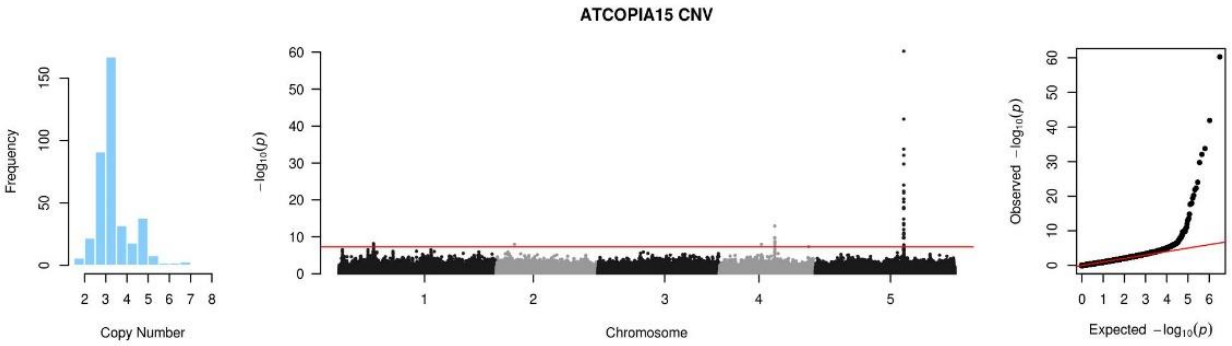

TE	GWAS associated interval	Leading SNP	Type of association	*P-value* Joined	*P-value* Worldwide	*P-value* Sweden	MAF	Candidate gene(s)
ATCOPIA15	Chr1:6691019-6693438	Chr1-6691020	*TRANS*	6.95E-09	1.07E-04	3.94E-07	0.264	*AT1G19340*
ATCOPIA15	Chr2:3543097-3543099	Chr2-3543098	*TRANS*	1.01E-08	2.68E-07	NA	0.056	.
ATCOPIA15	Chr4:8140855-8140857	Chr4-8140856	*TRANS*	1.01E-08	1.12E-06	NA	0.154	***AT4G14140 (MET2a)***
ATCOPIA15	Chr5:17086844-17108149	Chr5-17095071	*CIS*	5.36E-61	1.59E-29	3.92E-43	0.200	

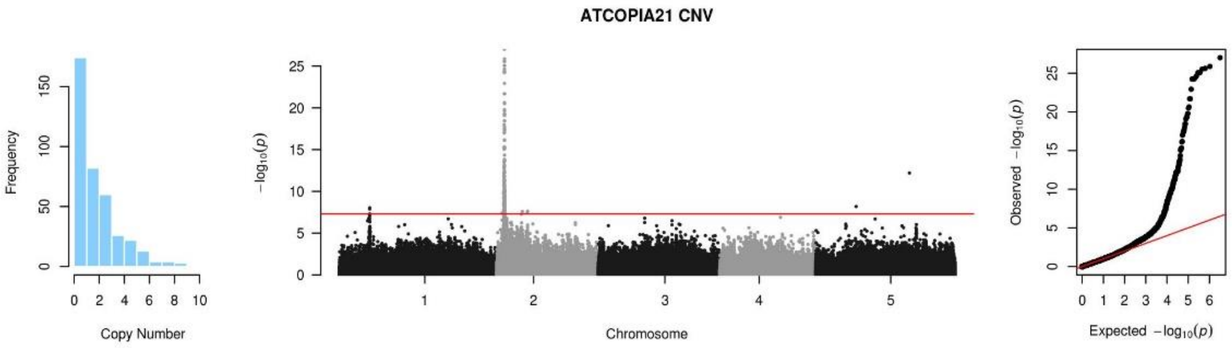

TE	GWAS associated interval	Leading SNP	Type of association	*P-value* Joined	*P-value* Worldwide	*P-value* Sweden	MAF	Candidate gene(s)
ATCOPIA21	Chr1:5907163-5932529	Chr1-5932528	*TRANS*	9.27E-09	1.07E-02	3.30E-07	0.097	*AT1G17240, AT1G17250, AT1G17255, AT1G17260, AT1G17270, AT1G17280, AT1G17285, AT1G17290, AT1G17300, AT1G17310, AT1G17330, AT1G17340, AT1G17345, AT1G17350*
ATCOPIA21	Chr2:1361817-1569513	Chr2-1545294	*TRANS*	1.00E-27	2.63E-11	2.25E-17	0.162	Centromere
ATCOPIA21	Chr2:5003212-5003214	Chr2-5003213	*TRANS*	2.53E-08	NA	4.52E-05	0.062	*AT2G12400, AT2G12405*
ATCOPIA21	Chr2:6027055-6027057	Chr2-6027056	*TRANS*	2.33E-08	NA	3.70E-06	0.079	*AT2G14210, AT2G14247, AT2G14255*
ATCOPIA21	Chr5:7834869-7834871	Chr5-7834870	*TRANS*	6.44E-09	2.00E-05	9.53E-05	0.156	*AT5G23230, AT5G23240, AT5G23250, AT5G23260, AT5G23270, AT5G23280*
ATCOPIA21	Chr5:18146702-18146704	Chr5-18146703	*CIS*	6.37E-13	3.74E-05	2.26E-10	0.341	

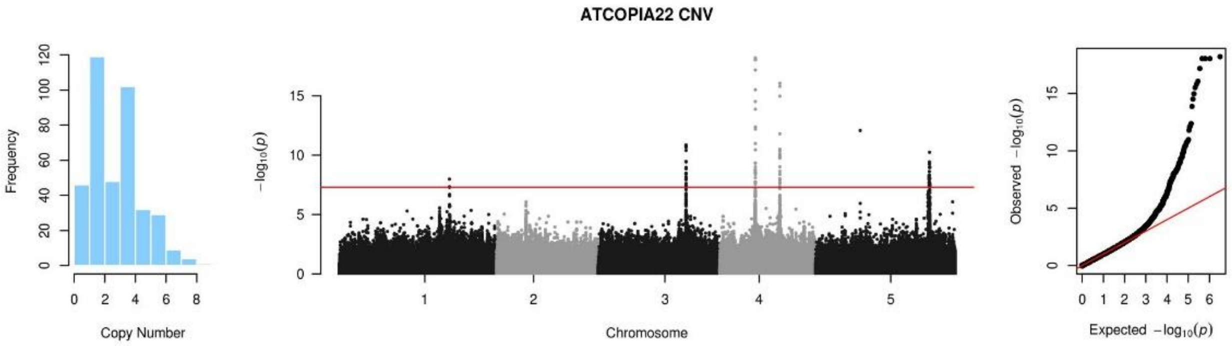

TE	GWAS associated interval	Leading SNP	Type of association	*P-value*			MAF	Candidate gene(s)
				Joined	Worldwide	Sweden		
ATCOPIA22	Chr1:21331281-21331283	Chr1-21331282	*TRANS*	1.03E-08	2.11E-05	5.79E-05	0.131	*AT1G57570, AT1G57580, AT1G57590, AT1G57600, AT1G57610, AT1G57613*
ATCOPIA22	Chr3:16986926-16997760	Chr3-16990821	*CIS*	1.56E-11	8.86E-10	3.02E-04	0.233	
ATCOPIA22	Chr4:6889588-6925403	Chr4-6914365	*CIS*	6.20E-19	7.33E-10	3.78E-11	0.415	
ATCOPIA22	Chr4:11674680-11689673	Chr4-11683009	*CIS*	8.60E-17	3.88E-10	5.46E-08	0.413	
ATCOPIA22	Chr5:8626824-8626826	Chr5-8626825	*CIS*	8.35E-13	6.06E-06	7.19E-09	0.321	
ATCOPIA22	Chr5:21983780-22083456	Chr5-22008321	*CIS*	3.64E-10	7.01E-04	3.74E-08	0.397	

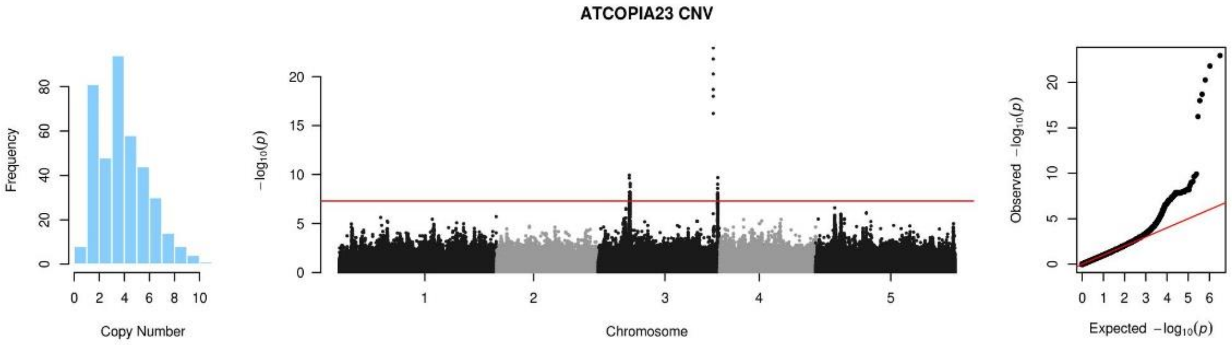

TE	GWAS associated interval	Leading SNP	Type of association	*P-value*			MAF	Candidate gene(s)
				Joined	Worldwide	Sweden		
ATCOPIA23	Chr3:6003034-6152323	Chr3-6009730	*CIS*	1.18E-10	NA	3.66E-10	0.428	
ATCOPIA23	Chr3:22233653-22236246	Chr3-22234997	*CIS*	1.13E-23	4.31E-13	5.84E-13	0.303	
ATCOPIA23	Chr3:23091858-23119620	Chr3-23095275	*CIS*	9.59E-10	2.32E-10	NA	0.387	

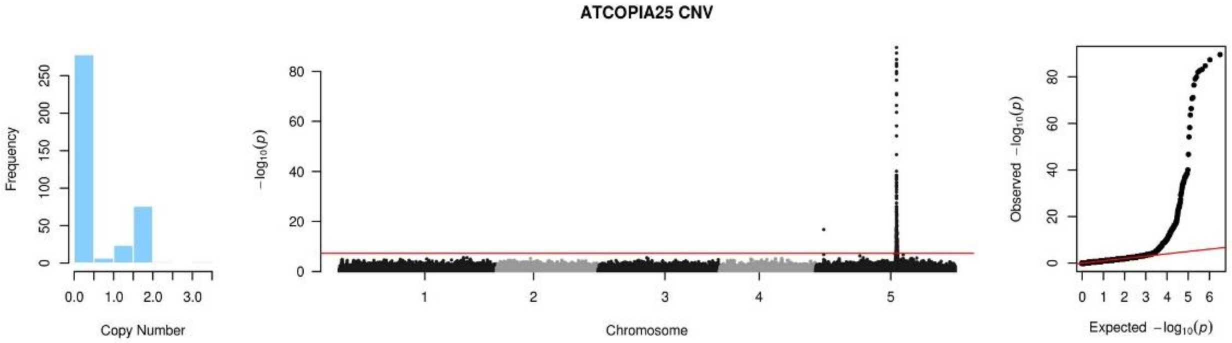

TE	GWAS associated interval	Leading SNP	Type of association	*P-value* Joined	Worldwide	Sweden	MAF	Candidate gene(s)
ATCOPIA25	Chr5:15631732-15722788	Chr5-1565233	*TRANS*	1.71E-17	2.75E-08	3.32E-11	0.387	*AT5G05240, AT5G05250, AT5G05260, AT5G05270, AT5G05280, AT5G05282, AT5G05290, AT5G05300, AT5G05310, AT5G05320*
ATCOPIA25	Chr5:1565232-1565234	Chr5-15536214	*TRANS*	4.87E-09	1.07E-08	1.13E-01	0.338	*AT5G38760, AT5G38770, AT5G38780, AT5G38790, AT5G38800, AT5G38810, AT5G38820, AT5G38830*
ATCOPIA25	Chr5:15534667-15536215	Chr5-15646847	*CIS*	2.96E-90	4.49E-49	2.52E-43	0.244	

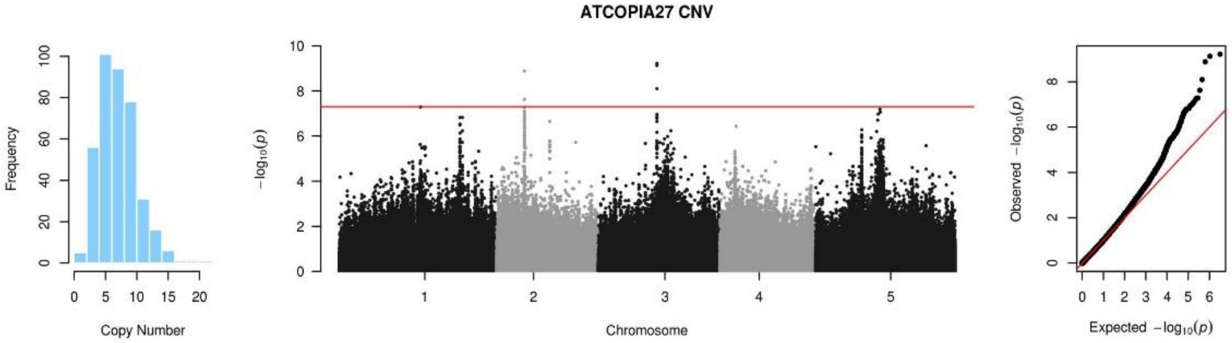

TE	GWAS associated interval	Leading SNP	Type of association	*P-value* Joined	Worldwide	Sweden	MAF	Candidate gene(s)
ATCOPIA27	Chr2:5395709-5399290	Chr2-5399289	*CIS*	1.30E-09	3.06E-04	3.54E-07	0.426	
ATCOPIA27	Chr3:11336905-11341663	Chr3-11341654	*CIS*	6.02E-10	NA	3.69E-08	0.364	

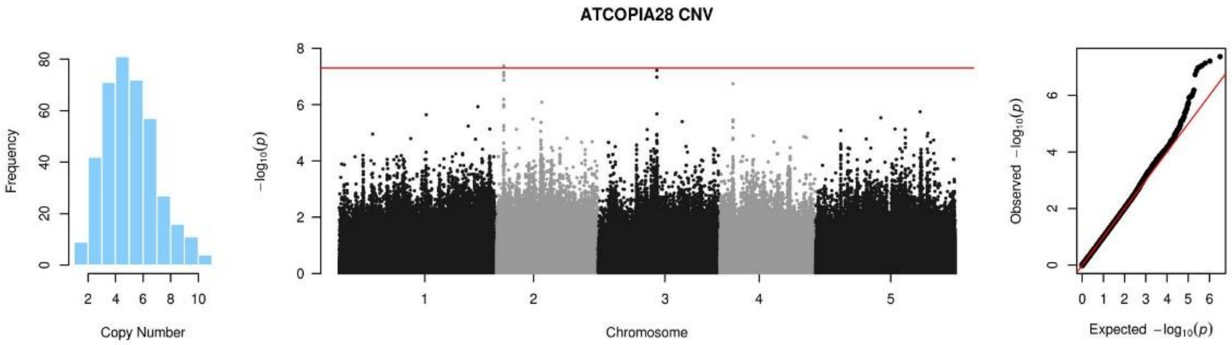

TE	GWAS associated interval	Leading SNP	Type of association	*P-value* Joined	Worldwide	Sweden	MAF	Candidate gene(s)

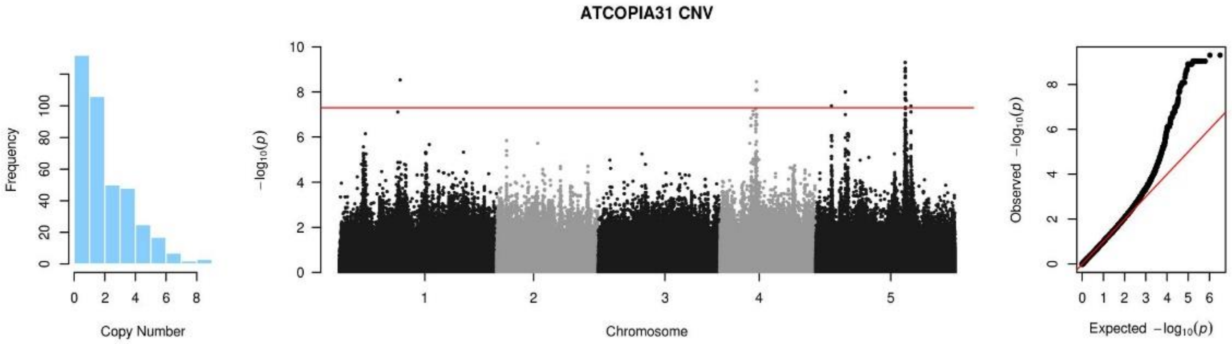

TE	GWAS associated interval	Leading SNP	Type of association	*P-value* Joined	Worldwide	Sweden	MAF	Candidate gene(s)
ATCOPIA31	Chr1:11792244-11792246	Chr1-11792245	*CIS*	2.93E-09	2.70E-04	1.90E-06	0.233	
ATCOPIA31	Chr4:7115170-7148336	Chr4-7115171	*TRANS*	8.21E-09	8.98E-04	NA	0.056	*AT4G11810, AT4G11820, AT4G11830, AT4G11840, AT4G11845, AT4G11850, AT4G11860, AT4G11870, AT4G11876, AT4G11880, AT4G11890, AT4G11900, AT4G11910, AT4G11911*
ATCOPIA31	Chr5:17518855-17518857	Chr5-5749637	*CIS*	1.00E-08	1.04E-04	8.20E-05	0.121	
ATCOPIA31	Chr5:5749636-5753383	Chr5-17518856	*TRANS*	2.39E-08	9.60E-05	9.55E-06	0.179	*AT5G43620/AT5G43630*

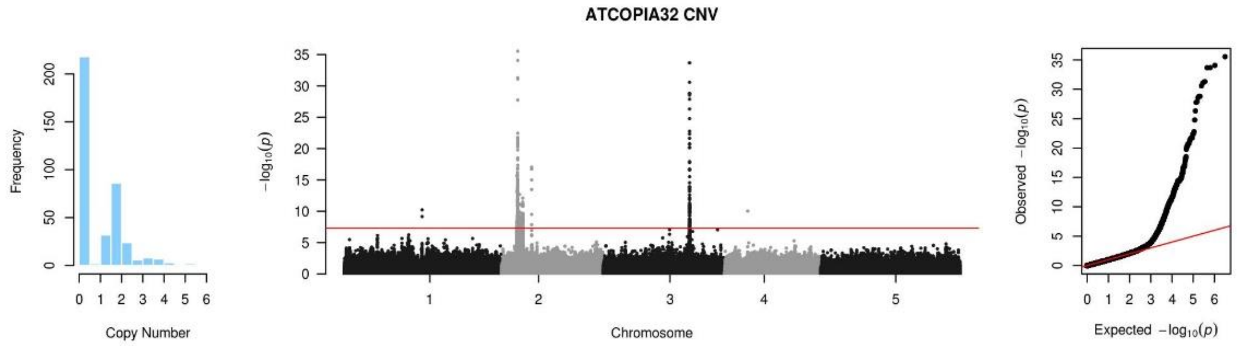

TE	GWAS associated interval	Leading SNP	Type of association	*P-value*			MAF	Candidate gene(s)
				Joined	Worldwide	Sweden		
ATCOPIA32	Chr1:15081095-15081108	Chr1-15081107	*TRANS*	5.86E-11	5.25E-07	4.82E-06	0.262	*AT1G40104*
ATCOPIA32	Chr2:2988052-3233924	Chr2-3165157	*CIS*	2.85E-36	4.63E-20	1.27E-19	0.213	
ATCOPIA32	Chr2:3912394-4142561	Chr2-4116428	*TRANS*	7.79E-13	6.01E-07	4.03E-10	0.149	*AT2G10260, AT2G10340, AT2G10440, AT2G10450, AT2G10535, AT2G10537, AT2G10550, AT2G10557, AT2G10560, AT2G10602,* ***AT2G10606 (AtMIR396a)****, AT2G10608*
ATCOPIA32	Chr2:5842521-5844665	Chr2-5844284	*CIS*	9.22E-18	3.28E-13	4.77E-05	0.141	
ATCOPIA32	Chr3:16653606-16706917	Chr3-16692698	*CIS*	2.05E-34	1.54E-15	1.28E-19	0.228	
ATCOPIA32	Chr4:4490149-4490151	Chr4-4490150	*TRANS*	9.43E-11	5.10E-06	1.29E-06	0.136	.

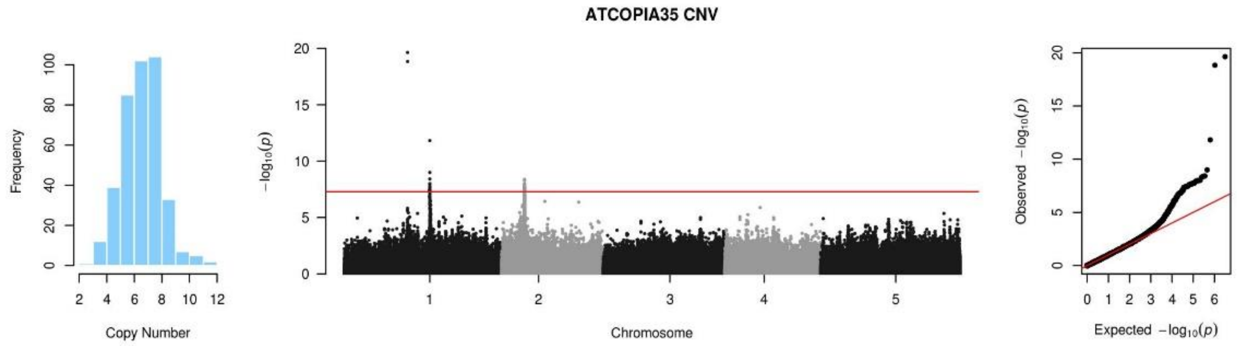

TE	GWAS associated interval	Leading SNP	Type of association	*P-value*			MAF	Candidate gene(s)
				Joined	Worldwide	Sweden		
ATCOPIA35	Chr1:12275476-12276017	Chr1-12275477	*CIS*	2.33E-20	4.01E-12	1.10E-08	0.238	
ATCOPIA35	Chr1:16569369-16574867	Chr1-16569613	*CIS*	1.54E-12	8.37E-08	5.06E-06	0.492	
ATCOPIA35	Chr2:4433215-4466730	Chr2-4460190	*CIS*	9.72E-09	3.94E-04	3.39E-07	0.267	

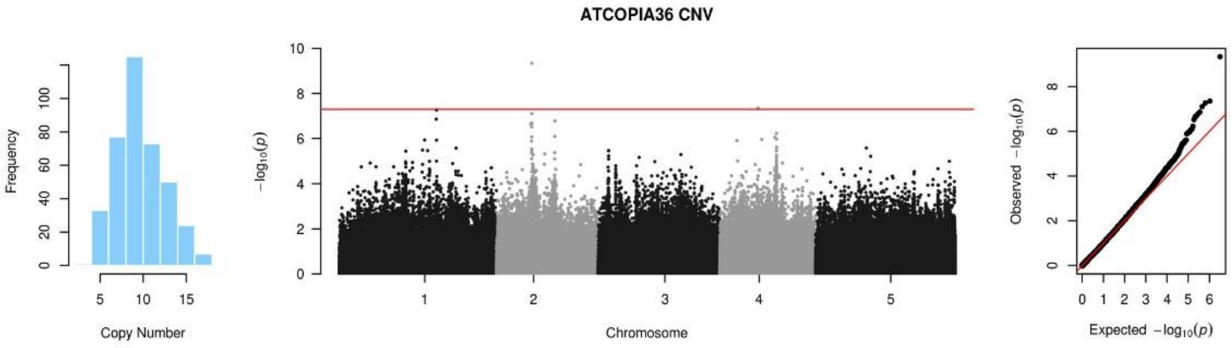

TE	GWAS associated interval	Leading SNP	Type of association	*P-value*			MAF	Candidate gene(s)
				Joined	Worldwide	Sweden		
ATCOPIA36	Chr2:6823369-6823371	Chr2-6823370	*CIS*	4.60E-10	1.09E-08	5.98E-04	0.141	

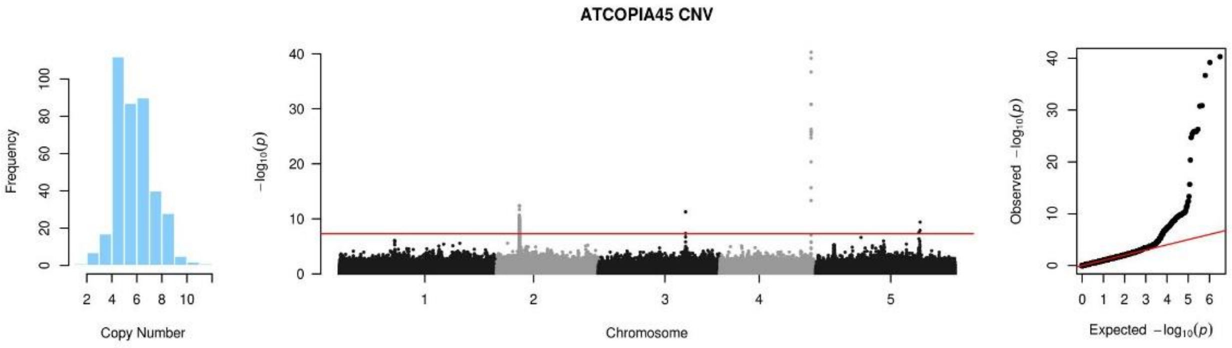

TE	GWAS associated interval	Leading SNP	Type of association	*P-value*			MAF	Candidate gene(s)
				Joined	Worldwide	Sweden		
ATCOPIA45	Chr2:4424480-4470623	Chr2-4448145	*CIS*	3.80E-13	2.07E-06	5.36E-11	0.326	
ATCOPIA45	Chr3:16887713-16888130	Chr3-16887714	*CIS*	5.06E-12	1.15E-07	1.03E-05	0.167	
ATCOPIA45	Chr4:17712213-17716723	Chr4-17714966	*CIS*	4.92E-41	4.04E-20	4.54E-22	0.385	
ATCOPIA45	Chr5:19992222-20214930	Chr5-20214929	*CIS*	3.72E-10	2.05E-06	3.82E-05	0.113	

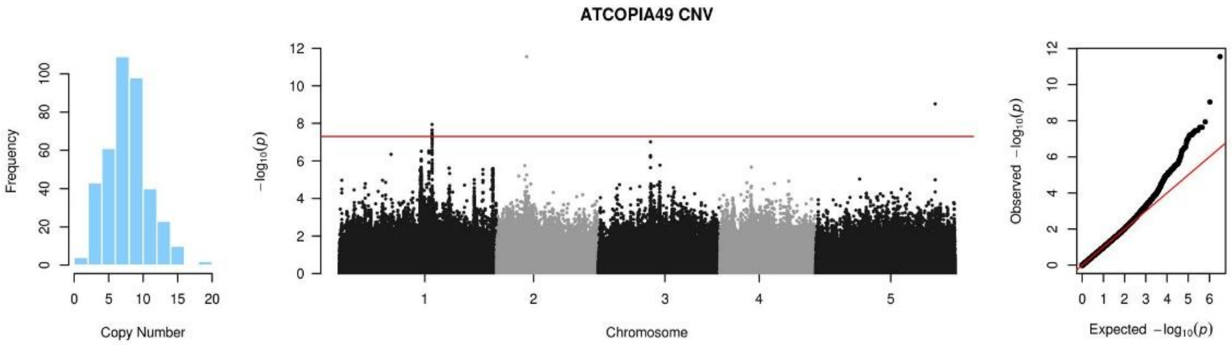

TE	GWAS associated interval	Leading SNP	Type of association	*P-value*			MAF	Candidate gene(s)
				Joined	Worldwide	Sweden		
ATCOPIA49	Chr1:17971317-17972346	Chr1-17971318	*TRANS*	2.30E-08	1.89E-02	1.90E-07	0.062	*AT1G48610, AT1G48620*
ATCOPIA49	Chr5:23137090-23137092	Chr5-23137091	*CIS*	9.10E-10	1.05E-06	9.76E-04	0.300	

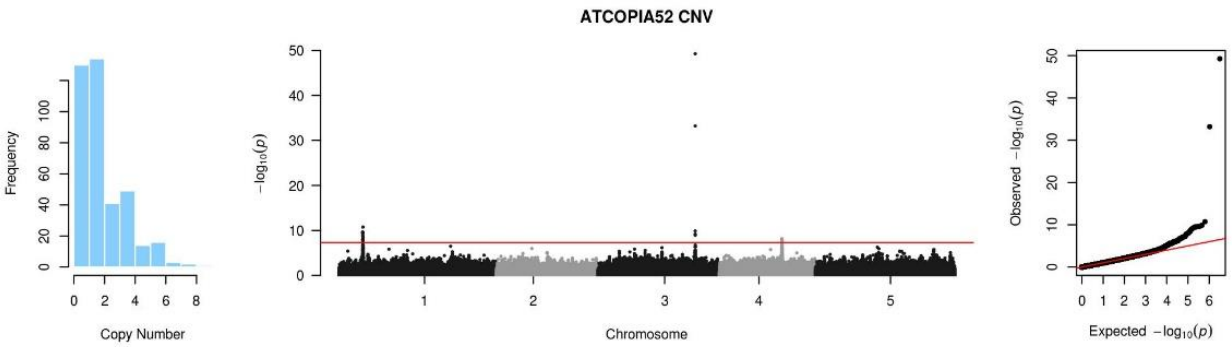

TE	GWAS associated interval	Leading SNP	Type of association	*P-value*			MAF	Candidate gene(s)
				Joined	Worldwide	Sweden		
ATCOPIA52	Chr1:4601427-4637435	Chr1-4608098	*CIS*	2.46E-10	NA	6.84E-10	0.223	
ATCOPIA52	Chr3:18791446-18794342	Chr3-18794341	*CIS*	5.44E-50	9.81E-29	3.49E-23	0.341	
ATCOPIA52	Chr4:12087454-12124406	Chr4-12087455	*CIS*	6.92E-09	8.35E-06	2.69E-05	0.169	

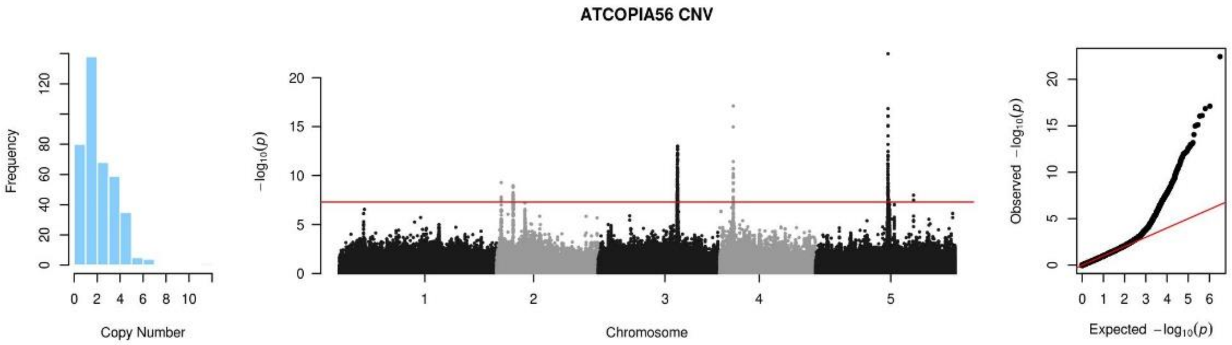

TE	GWAS associated interval	Leading SNP	Type of association	*P-value*			MAF	Candidate gene(s)
				Joined	Worldwide	Sweden		
ATCOPIA56	Chr2:926086-928373	Chr2-928372	*CIS*	1.59E-08	NA	1.59E-08	0.120	
ATCOPIA56	Chr2:3234681-3237292	Chr2-3234682	*TRANS*	1.08E-09	7.71E-07	6.15E-04	0.238	*AT2G07742, AT2G07749, AT2G07771, AT2G07773, AT2G07776*
ATCOPIA56	Chr3:15250312-15250314	Chr3-15250313	*TRANS*	7.72E-09	8.45E-03	1.25E-07	0.126	*AT3G43300*
ATCOPIA56	Chr4:2638412-2651109	Chr4-2644995	*CIS*	7.81E-18	6.95E-11	9.70E-10	0.436	
ATCOPIA56	Chr5:13954062-14040109	Chr5-13998970	*CIS*	3.65E-23	8.03E-15	2.19E-10	0.369	
ATCOPIA56	Chr5:14126672-14136469	Chr5-14126673	*TRANS*	2.91E-08	7.05E-04	2.99E-06	0.238	*AT5G35970, AT5G35980, AT5G35995, AT5G36000, AT5G36001, AT5G36002*
ATCOPIA56	Chr5:18934190-18934192	Chr5-18934191	*TRANS*	9.89E-09	NA	5.27E-07	0.128	*AT5G46640, AT5G46650, AT5G46660, AT5G46670, AT5G46680*

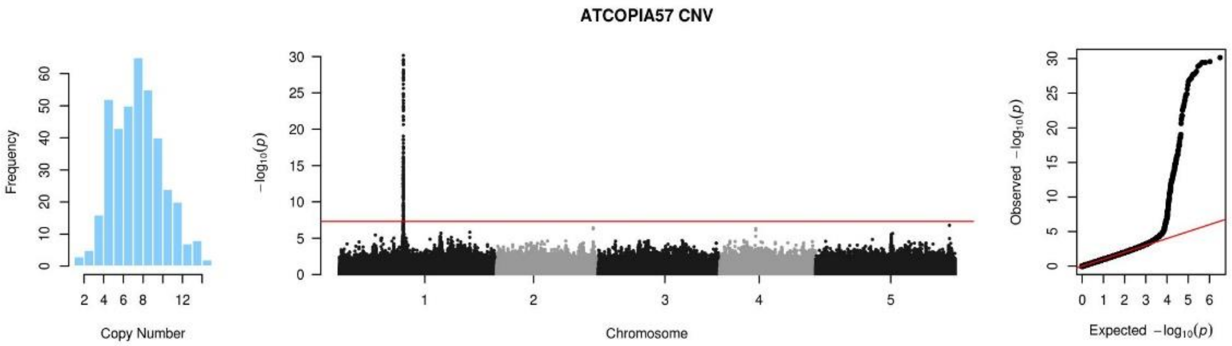

TE	GWAS associated interval	Leading SNP	Type of association	*P-value*			MAF	Candidate gene(s)
				Joined	Worldwide	Sweden		
ATCOPIA57	Chr1:12397425-12419360	Chr1-12400225	*CIS*	7.06E-31	4.24E-20	6.40E-12	0.369	

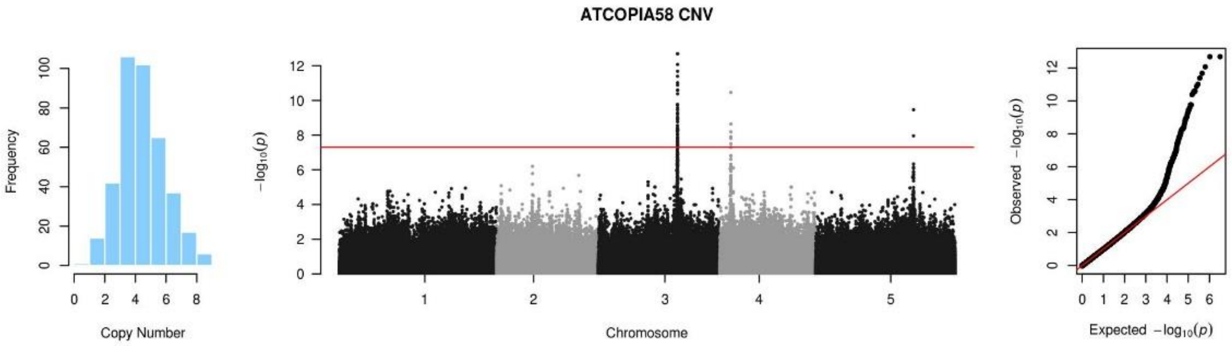

TE	GWAS associated interval	Leading SNP	Type of association	*P-value*			MAF	Candidate gene(s)
				Joined	Worldwide	Sweden		
ATCOPIA58	Chr3:15297177-15337326	Chr3-15333805	*CIS*	1.73E-10	1.09E-05	9.17E-07	0.264	
ATCOPIA58	Chr4:2196868-2204565	Chr4-2203315	*CIS*	2.29E-09	5.55E-03	1.06E-09	0.351	
ATCOPIA58	Chr5:18930571-18937598	Chr5-18937597	*CIS*	3.45E-10	NA	4.06E-11	0.295	

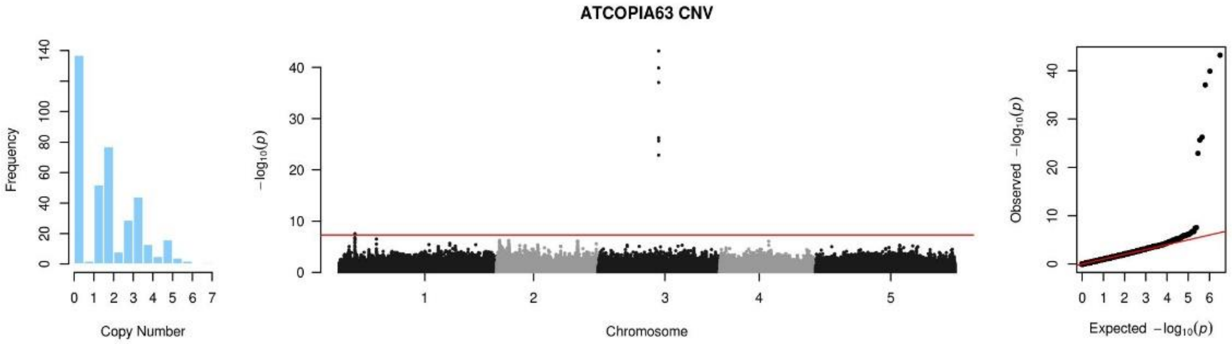

TE	GWAS associated interval	Leading SNP	Type of association	*P-value*			MAF	Candidate gene(s)
				Joined	Worldwide	Sweden		
ATCOPIA63	Chr1:3070484-3070486	Chr1-3070485	*TRANS*	2.75E-08	7.88E-06	NA	0.054	*AT1G09480, AT1G09483, AT1G09490, AT1G09500, AT1G09510, AT1G09520, AT1G09530*
ATCOPIA63	Chr3:11665988-11669337	Chr3-11668694	*CIS*	6.44E-44	3.05E-27	3.27E-22	0.367	

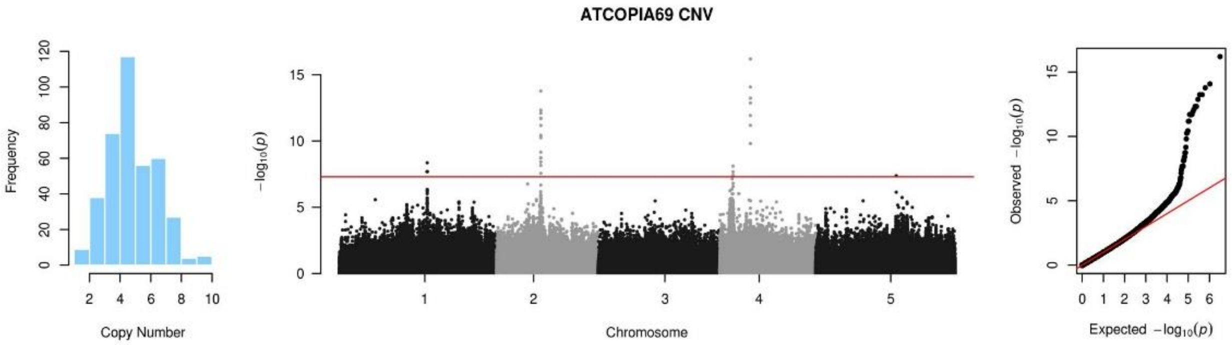

TE	GWAS associated interval	Leading SNP	Type of association	*P-value*			MAF	Candidate gene(s)
				Joined	Worldwide	Sweden		
ATCOPIA69	Chr1:17021538-17021715	Chr1-17021714	*TRANS*	4.39E-09	6.77E-07	NA	0.079	*AT1G45000, AT1G45010, AT1G45015, AT1G45050*
ATCOPIA69	Chr2:8562490-8566582	Chr2-8566517	*CIS*	1.68E-14	6.79E-09	1.92E-06	0.341	
ATCOPIA69	Chr4:2603653-2604070	Chr4-2604069	*CIS*	7.85E-09	5.67E-04	2.35E-07	0.185	
ATCOPIA69	Chr4:5967301-5969646	Chr4-5969528	*CIS*	6.40E-17	6.19E-12	4.63E-07	0.346	

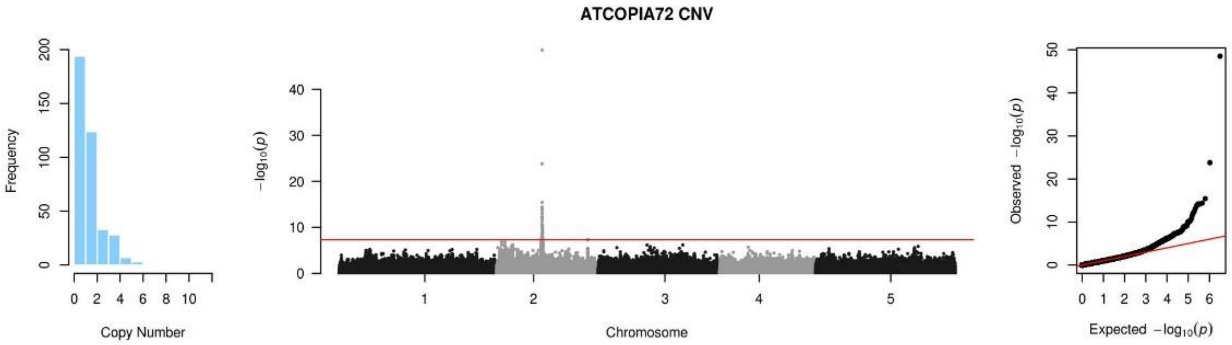

TE	GWAS associated interval	Leading SNP	Type of association	*P-value*			MAF	Candidate gene(s)
				Joined	Worldwide	Sweden		
ATCOPIA72	Chr2:8780704-8939446	Chr2-8820365	*CIS*	3.03E-49	6.58E-25	3.06E-24	0.474	

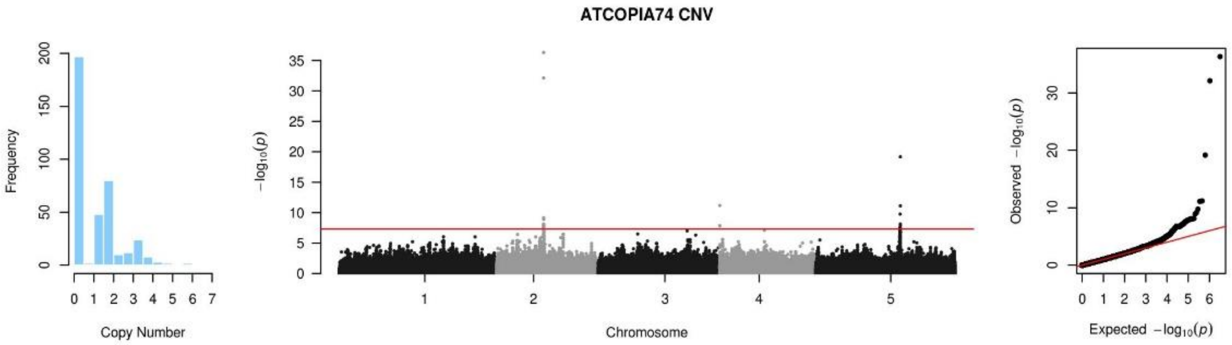

TE	GWAS associated interval	Leading SNP	Type of association	*P-value*			MAF	Candidate gene(s)
				Joined	Worldwide	Sweden		
ATCOPIA74	Chr2:9096957-9167689	Chr2-9124731	*CIS*	4.93E-37	4.19E-20	6.36E-19	0.438	
ATCOPIA74	Chr4:30568-41778	Chr4-41777	*TRANS*	6.56E-12	2.39E-07	2.50E-05	0.262	*AT4G00060, AT4G00070, AT4G00080, AT4G00085, AT4G00090, AT4G00100, AT4G00110, AT4G00120, AT4G00124, AT4G00130, AT4G00140*
ATCOPIA74	Chr5:16320093-16398302	Chr5-16389345	*CIS*	6.89E-20	1.13E-15	NA	0.090	

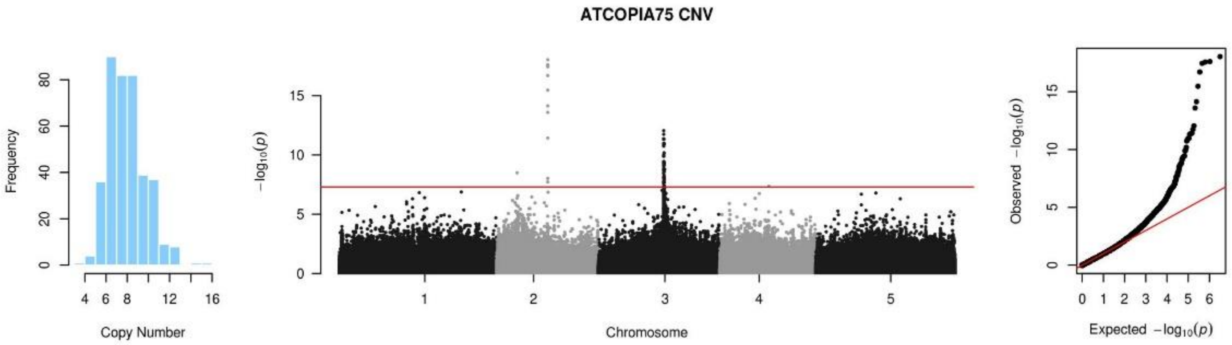

TE	GWAS associated interval	Leading SNP	Type of association	*P-value*			MAF	Candidate gene(s)
				Joined	Worldwide	Sweden		
ATCOPIA75	Chr2:3989860-3989862	Chr2-3989861	*TRANS*	3.13E-09	2.98E-09	8.57E-02	0.238	*AT2G10340*
ATCOPIA75	Chr2:9928878-9932430	Chr2-9930929	*CIS*	9.30E-19	1.17E-10	1.92E-10	0.477	
ATCOPIA75	Chr3:12834786-12834788	Chr3-12834787	*TRANS*	1.59E-09	9.04E-06	2.06E-06	0.338	.

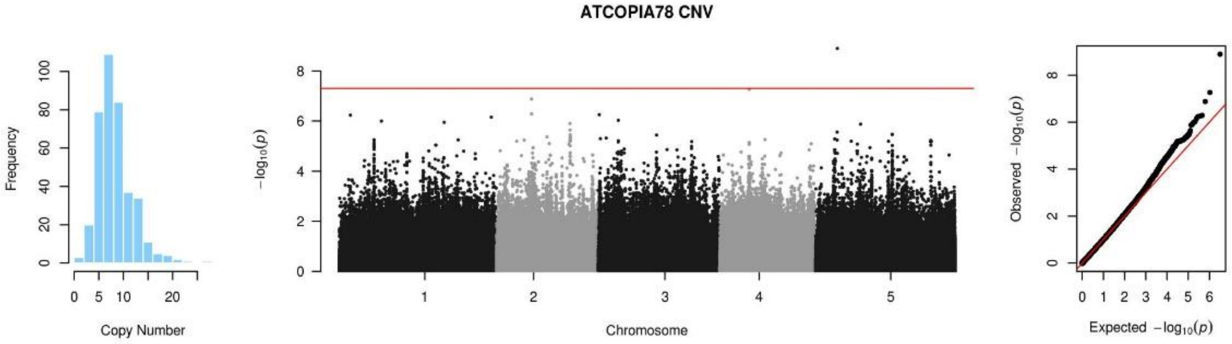

TE	GWAS associated interval	Leading SNP	Type of association	*P-value*			MAF	Candidate gene(s)
				Joined	Worldwide	Sweden		
ATCOPIA78	Chr5:4188262-4188264	Chr5-4188263	*TRANS*	1.27E-09	3.94E-10	NA	0.051	*AT5G13160, AT5G13170, AT5G13180*

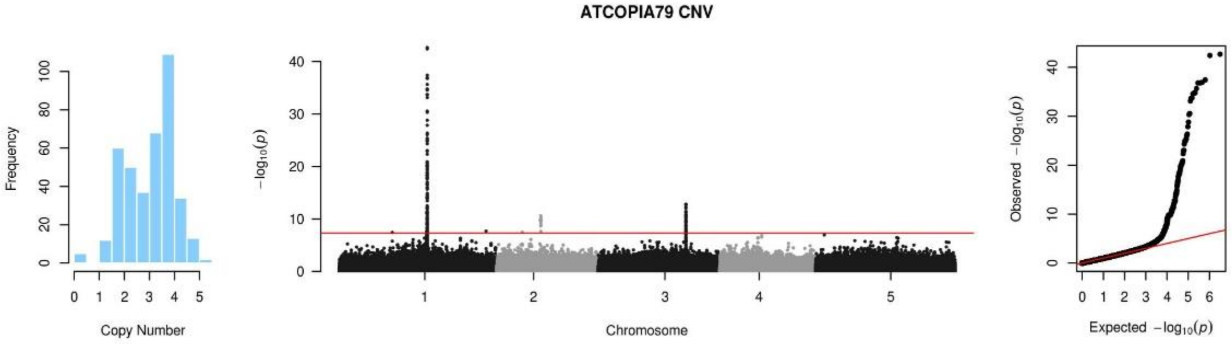

TE	GWAS associated interval	Leading SNP	Type of association	*P-value*			MAF	Candidate gene(s)
				Joined	Worldwide	Sweden		
ATCOPIA79	Chr1:16980013-17031219	Chr1-17025377	*CIS*	3.85E-43	1.93E-23	2.19E-23	0.292	
ATCOPIA79	Chr1:28424371-28424373	Chr1-28424372	*TRANS*	2.10E-08	1.14E-07	NA	0.118	*AT1G75660, AT1G75670, AT1G75680, AT1G75690, AT1G75700, AT1G75710, AT1G75717*
ATCOPIA79	Chr2:8562348-8566414	Chr2-8564058	*TRANS*	2.97E-11	1.65E-06	NA	0.056	*AT2G19820, AT2G19830, AT2G19850, AT2G19860, AT2G19870*
ATCOPIA79	Chr3:16954379-16964030	Chr3-16960376	*CIS*	1.73E-10	1.90E-05	1.93E-07	0.259	

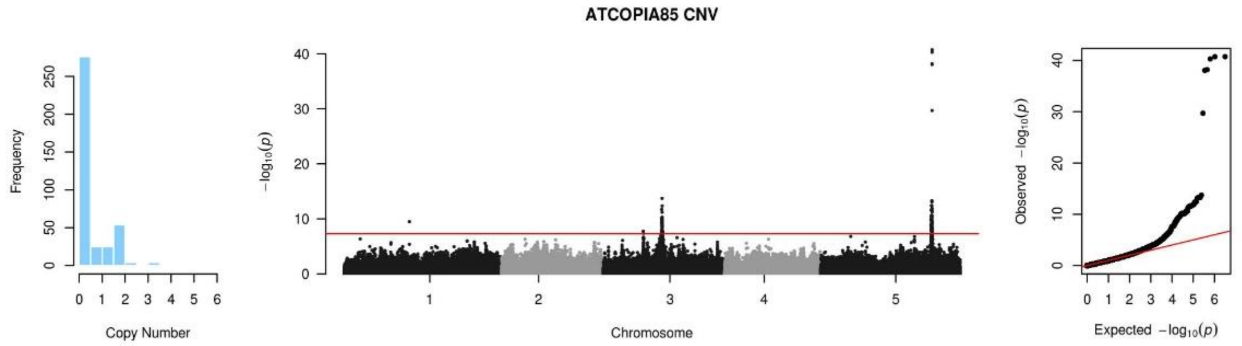

TE	GWAS associated interval	Leading SNP	Type of association	*P-value*			MAF	Candidate gene(s)
				Joined	Worldwide	Sweden		
ATCOPIA85	Chr1:12622354-12622356	Chr1-12622355	*CIS*	3.18E-10	NA	2.16E-08	0.121	
ATCOPIA85	Chr3:7751220-7751222	Chr3-7751221	*TRANS*	1.63E-08	3.56E-08	NA	0.077	*AT3G21970, AT3G21980, AT3G21990, AT3G22000, AT3G22010, AT3G22020, AT3G22022, AT3G22030*
ATCOPIA85	Chr3:11289786-11522466	Chr3-11350047	*TRANS*	1.52E-10	NA	6.32E-18	0.146	*AT3G29590, AT3G29636, AT3G29410, AT3G29644, AT3G29639, AT3G29638, AT3G29635, AT3G29633, AT3G29630, AT3G29390, AT3G29580, AT3G29575, AT3G29570, AT3G29560, AT3G29450, AT3G29431, AT3G29430, AT3G29400*
ATCOPIA85	Chr5:21392325-21611278	Chr5-21545142	*CIS*	1.88E-41	5.91E-12	5.81E-61	0.154	

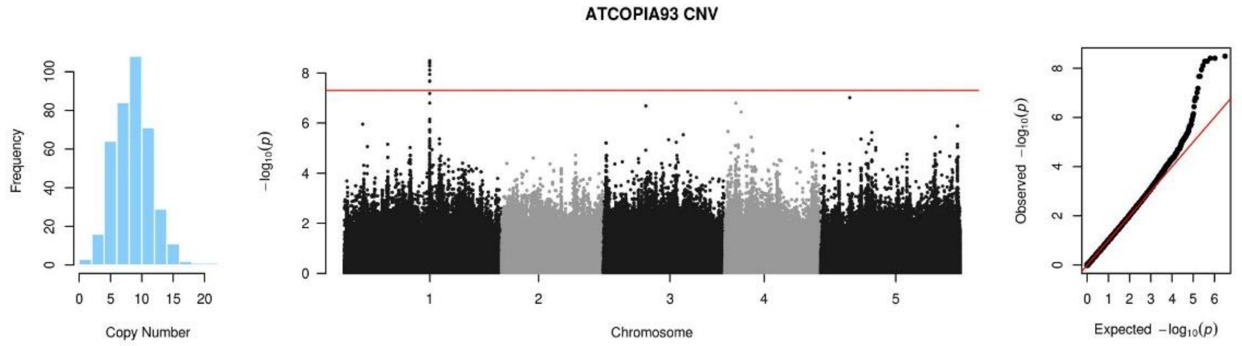

TE	GWAS associated interval	Leading SNP	Type of association	*P-value*			MAF	Candidate gene(s)
				Joined	Worldwide	Sweden		
ATCOPIA93	Chr1:16549768-16554098	Chr1-16549829	*CIS*	5.20E-09	1.29E-05	6.58E-05	0.413	

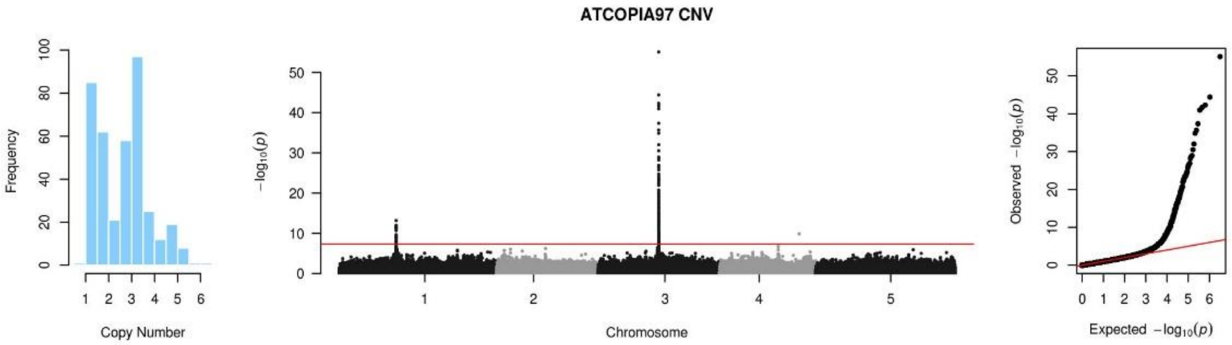

TE	GWAS associated interval	Leading SNP	Type of association	*P-value* Joined	Worldwide	Sweden	MAF	Candidate gene(s)
ATCOPIA97	Chr1:11011579-11013340	Chr1-11012665	*CIS*	2.28E-10	5.22E-05	4.04E-06	0.228	
ATCOPIA97	Chr3:11670224-11717607	Chr3-11680834	*CIS*	7.99E-56	6.09E-26	4.23E-33	0.410	
ATCOPIA97	Chr4:15398043-15398045	Chr4-15398044	*TRANS*	1.31E-10	2.46E-04	4.79E-09	0.279	*AT4G31830*

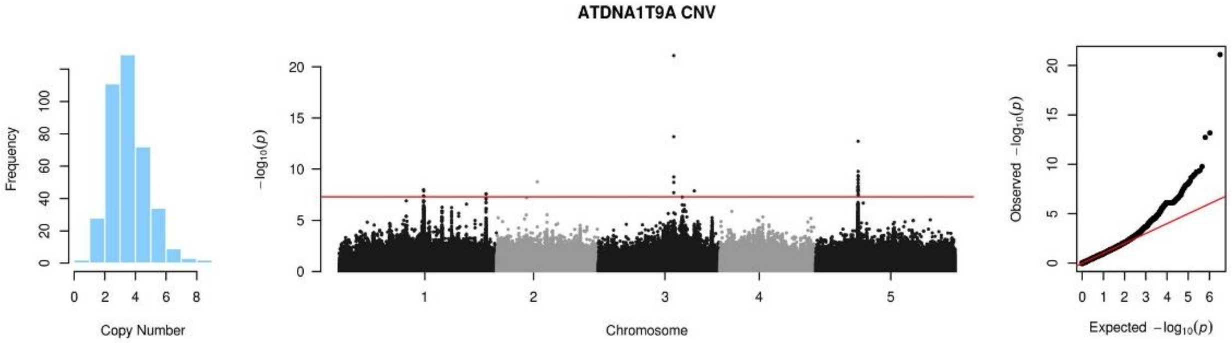

TE	GWAS associated interval	Leading SNP	Type of association	*P-value* Joined	Worldwide	Sweden	MAF	Candidate gene(s)
ATDNA1T9A	Chr1:16343965-16361551	Chr1-16343966	*CIS*	1.02E-08	2.57E-07	1.56E-03	0.169	*AT1G75660, AT1G75670, AT1G75680, AT1G75690, AT1G75700, AT1G75710*
ATDNA1T9A	Chr1:28419353-28419375	Chr1-28419354	*TRANS*	2.62E-08	7.92E-05	2.22E-05	0.238	
ATDNA1T9A	Chr2:7907427-7907429	Chr2-7907428	*TRANS*	1.70E-09	NA	3.61E-07	0.172	***AT2TE32765 (VANDAL16)***
ATDNA1T9A	Chr3:14600022-14603445	Chr3-14603444	*CIS*	8.11E-22	1.93E-12	1.39E-10	0.441	
ATDNA1T9A	Chr3:18568631-18568633	Chr3-18568632	*TRANS*	1.35E-08	NA	1.67E-07	0.085	*AT3G50060, AT3G50070, AT3G50080, AT3G50090, AT3G50100*
ATDNA1T9A	Chr5:8186376-8212656	Chr5-8206873	*CIS*	1.89E-13	5.03E-09	2.30E-06	0.256	

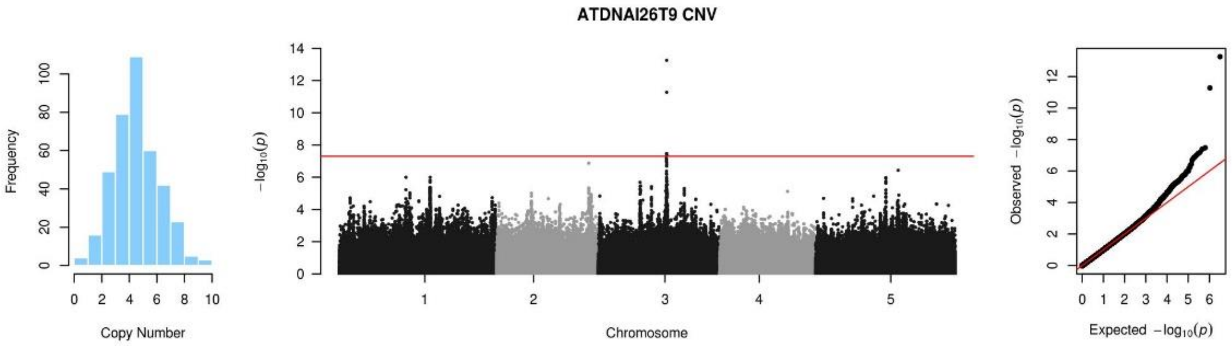

TE	GWAS associated interval	Leading SNP	Type of association	*P-value* Joined	Worldwide	Sweden	MAF	Candidate gene(s)
ATDNAI26T9	Chr3:13244967-13245592	Chr3-13244968	*CIS*	5.53E-14	1.35E-07	3.18E-08	0.049	

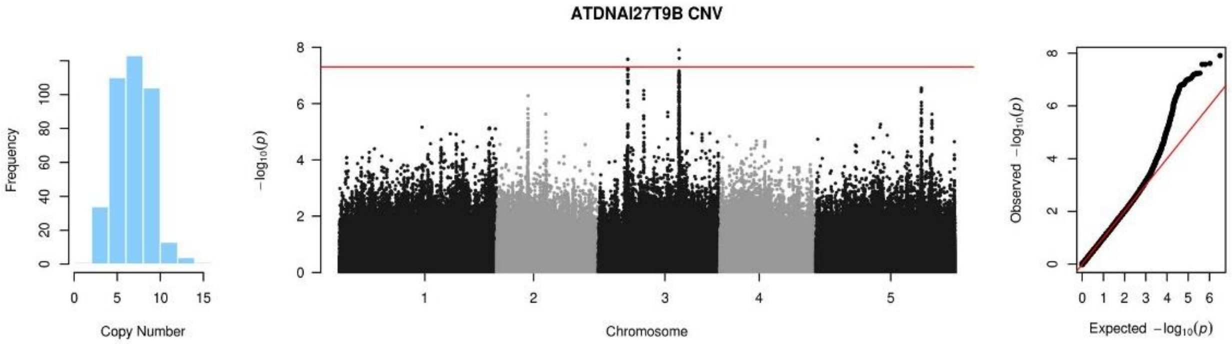

TE	GWAS associated interval	Leading SNP	Type of association	*P-value* Joined	Worldwide	Sweden	MAF	Candidate gene(s)
ATDNAI27T9B	Chr3:15617514-15642531	Chr3-5699617	*TRANS*	2.67E-08	9.60E-07	NA	0.069	*AT3G16700, AT3G16710, AT3G16712, AT3G16720, AT3G16730, AT3G16740, AT3G16750, AT3G16760, AT3G16770, AT3G16780, AT3G16785*
ATDNAI27T9B	Chr3:5699616-5707384	Chr3-15617515	*CIS*	1.24E-08	3.55E-05	1.92E-04	0.326	

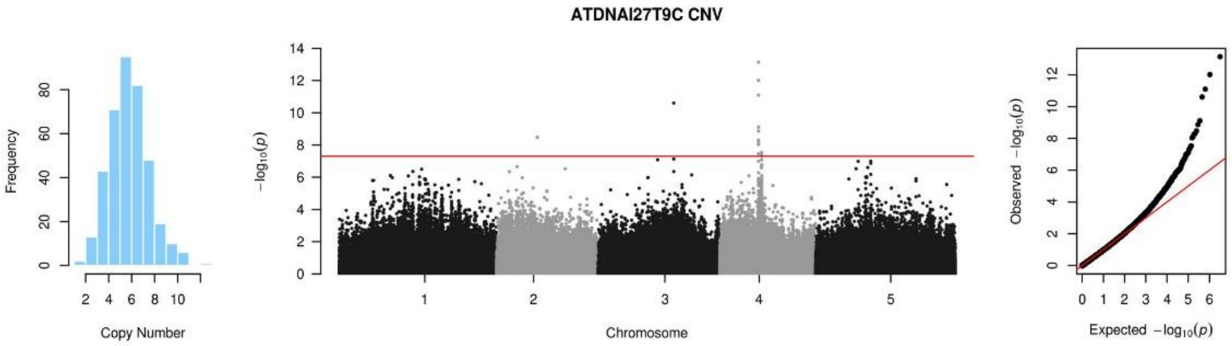

TE	GWAS associated interval	Leading SNP	Type of association	*P-value*			MAF	Candidate gene(s)
				Joined	Worldwide	Sweden		
ATDNAI27T9C	Chr2:7907427-7907429	Chr2-7907428	*TRANS*	3.32E-09	NA	5.06E-07	0.172	*AT2G18160, AT2G18162, AT2G18170, AT2G18180, AT2G18190*
ATDNAI27T9C	Chr3:14600022-14600024	Chr3-14600023	*TRANS*	2.47E-11	4.57E-06	6.48E-07	0.285	*AT3G42473, AT3G42475*
ATDNAI27T9C	Chr4:7524860-7526558	Chr4-7526557	*CIS*	7.75E-10	1.33E-07	4.76E-05	0.008	
ATDNAI27T9C	Chr4:8141395-8141397	Chr4-8141396	*TRANS*	2.92E-08	3.21E-05	6.42E-05	0.177	***AT4G14140 (MET2a)***

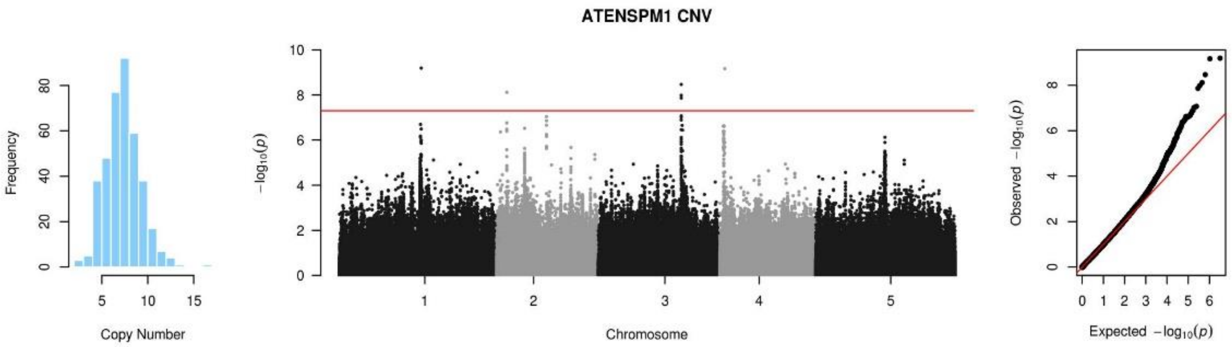

TE	GWAS associated interval	Leading SNP	Type of association	*P-value*			MAF	Candidate gene(s)
				Joined	Worldwide	Sweden		
ATENSPM1	Chr1:15867785-15867787	Chr1-15867786	*CIS*	6.43E-10	1.88E-05	1.44E-05	0.426	
ATENSPM1	Chr2:2006442-2006444	Chr2-2006443	*TRANS*	7.60E-09	1.88E-02	7.48E-07	0.121	*AT2G05500, AT2G05510*
ATENSPM1	Chr3:16050194-16050498	Chr3-16050497	*TRANS*	3.41E-09	NA	4.11E-08	0.167	*AT3G44400*
ATENSPM1	Chr4:1022686-1022688	Chr4-1022687	*CIS*	6.78E-10	1.76E-06	1.24E-04	0.313	

ATENSPM2 CNV

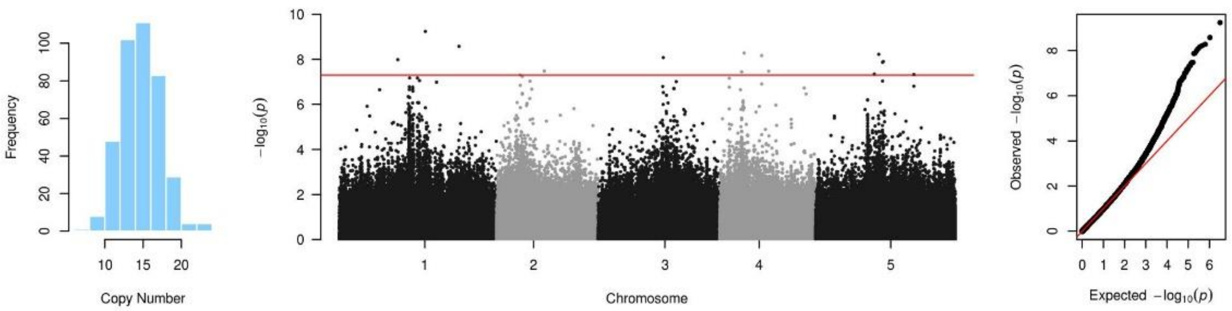

TE	GWAS associated interval	Leading SNP	Type of association	*P-value* Joined	Worldwide	Sweden	MAF	Candidate gene(s)
ATENSPM2	Chr1:16675002-16675004	Chr1-16675003	*TRANS*	5.75E-10	5.97E-07	5.20E-03	0.359	***AT1G43950 (ARF23)***
ATENSPM2	Chr1:23181917-23181919	Chr1-23181918	*TRANS*	2.64E-09	1.56E-07	4.40E-02	0.195	*AT1G62570, AT1G62580, AT1G62590, AT1G62600, AT1G62610, AT1G62620, AT1G62630*
ATENSPM2	Chr3:12566674-12566676	Chr3-12566675	*TRANS*	8.39E-09	4.71E-07	NA	0.218	.
ATENSPM2	Chr4:4767946-4767948	Chr4-4767947	*TRANS*	5.25E-09	9.69E-08	2.00E-02	0.321	.
ATENSPM2	Chr4:8148250-8148252	Chr4-8148251	*TRANS*	6.85E-09	1.27E-08	NA	0.108	***AT4G14140 (MET2a)***
ATENSPM2	Chr5:12205883-12205885	Chr5-12205884	*TRANS*	5.99E-09	3.63E-08	1.42E-01	0.282	.
ATENSPM2	Chr5:12947984-13085062	Chr5-13085061	*TRANS*	1.26E-08	1.16E-06	4.61E-03	0.287	*AT5G34780, AT5G34828, AT5G34829, AT5G34830, AT5G34869, AT5G34871*

ATENSPM3 CNV

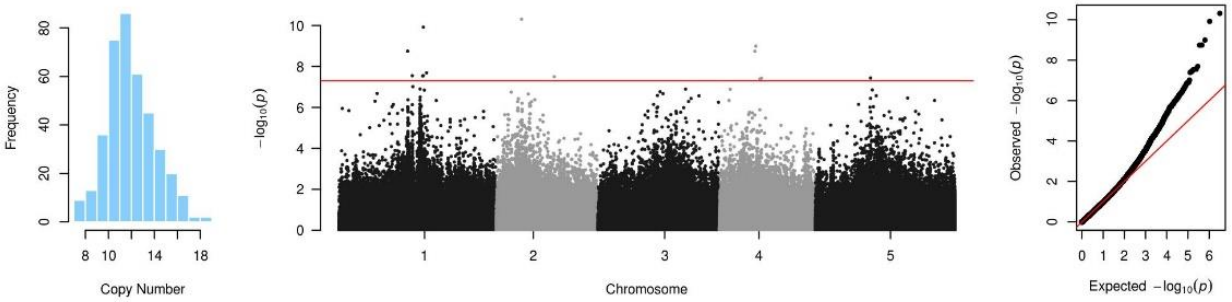

TE	GWAS associated interval	Leading SNP	Type of association	*P-value* Joined	Worldwide	Sweden	MAF	Candidate gene(s)
ATENSPM3	Chr1:13288207-13288209	Chr1-13288208	*TRANS*	1.80E-09	5.09E-09	NA	0.277	*AT1G35780*
ATENSPM3	Chr1:14186622-14186624	Chr1-14186623	*TRANS*	2.81E-08	3.97E-07	1.79E-02	0.377	*AT1G37150, AT1G37162*
ATENSPM3	Chr1:16182880-16182882	Chr1-16182881	*TRANS*	2.85E-08	6.45E-07	4.20E-03	0.423	*AT1G43040*
ATENSPM3	Chr1:16344111-16344119	Chr1-16344112	*TRANS*	1.22E-10	2.92E-12	7.14E-01	0.310	*AT1G43310*
ATENSPM3	Chr1:16962663-16962665	Chr1-16962664	*TRANS*	2.04E-08	1.51E-07	6.56E-03	0.226	*AT1G44900*
ATENSPM3	Chr2:4906414-4906416	Chr2-4906415	*CIS*	4.88E-11	2.31E-07	1.79E-03	0.374	
ATENSPM3	Chr4:6880307-6880309	Chr4-6880308	*TRANS*	1.82E-09	5.90E-09	3.96E-02	0.479	*AT4G11290, AT4G11300, AT4G11310, AT4G11320*
ATENSPM3	Chr4:7062242-7062244	Chr4-7062243	*TRANS*	1.02E-09	4.21E-09	NA	0.223	*AT4G11670, AT4G11680, AT4G11690, AT4G11700, AT4G11720, AT4G11730, AT4G11740*

ATENSPM5 CNV

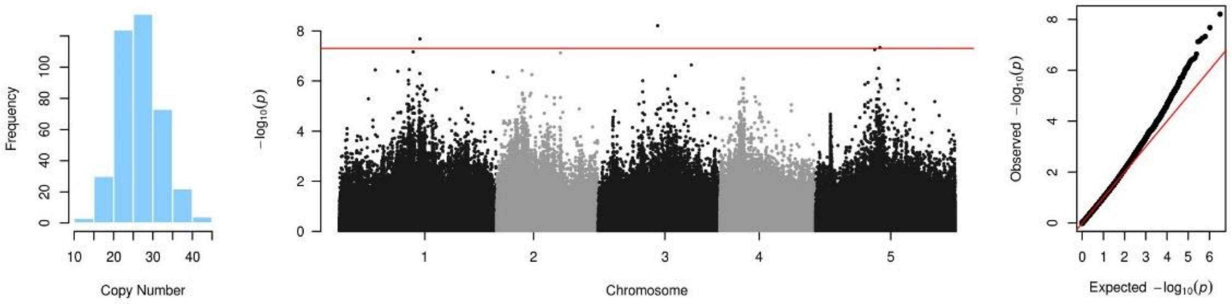

TE	GWAS associated interval	Leading SNP	Type of association	*P-value* Joined	Worldwide	Sweden	MAF	Candidate gene(s)
ATENSPM5	Chr1:15612278-15612280	Chr1-15612279	*TRANS*	2.10E-08	3.11E-08	NA	0.187	*AT1G41830*
ATENSPM5	Chr3:11495299-11495301	Chr3-11495300	*TRANS*	6.22E-09	8.24E-10	NA	0.228	*AT3G29644*

ATGP2 CNV

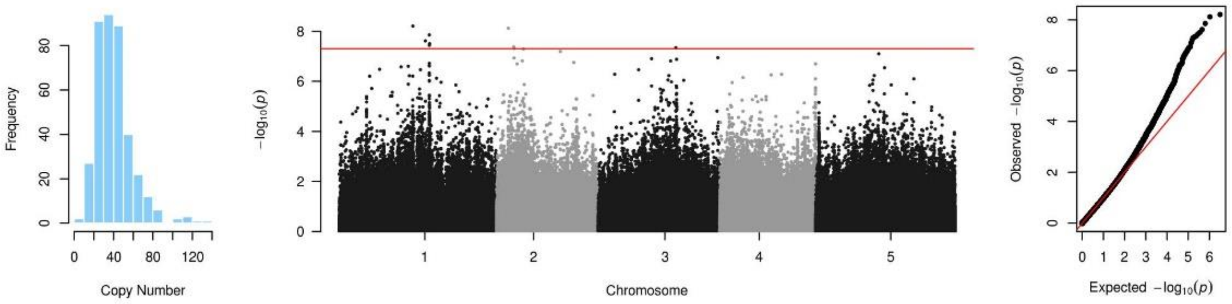

TE	GWAS associated interval	Leading SNP	Type of association	*P-value* Joined	Worldwide	Sweden	MAF	Candidate gene(s)
ATGP2	Chr1:14263097-14263099	Chr1-14263098	*TRANS*	6.21E-09	1.25E-08	NA	0.244	.
ATGP2	Chr1:16675002-16675004	Chr1-16675003	*TRANS*	2.42E-08	6.62E-09	2.84E-01	0.359	***AT1G43950 (ARF23)***
ATGP2	Chr1:17440198-17440200	Chr1-17440199	*TRANS*	1.40E-08	8.34E-07	8.78E-04	0.064	*AT1G47497, AT1G47500, AT1G47510, AT1G47497, AT1G47500, AT1G47510*
ATGP2	Chr2:2290684-2290686	Chr2-2290685	*TRANS*	7.61E-09	2.78E-06	2.23E-03	0.295	*AT2G05940*

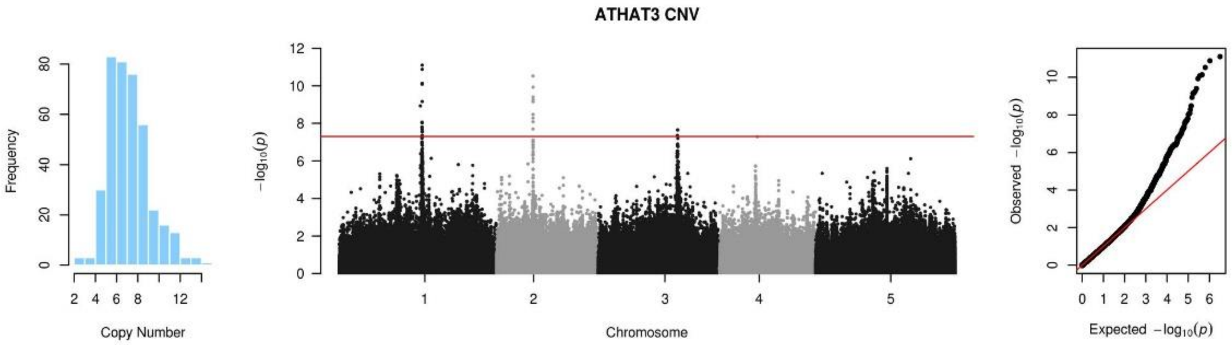

TE	GWAS associated interval	Leading SNP	Type of association	*P-value* Joined	Worldwide	Sweden	MAF	Candidate gene(s)
ATHAT3	Chr1:16036245-16036886	Chr1-16036246	*TRANS*	8.67E-09	6.36E-06	4.88E-05	0.282	*AT1G42680*
ATHAT3	Chr1:16070455-16094668	Chr1-16071288	*CIS*	7.91E-12	5.48E-11	NA	0.121	
ATHAT3	Chr2:7066692-7073458	Chr2-7067625	*CIS*	2.98E-11	5.18E-06	1.21E-06	0.364	
ATHAT3	Chr3:15351283-15351287	Chr3-15351284	*TRANS*	2.23E-08	NA	1.09E-07	0.108	*AT3G43420, AT3G43430, AT3G43431, AT3G43432*

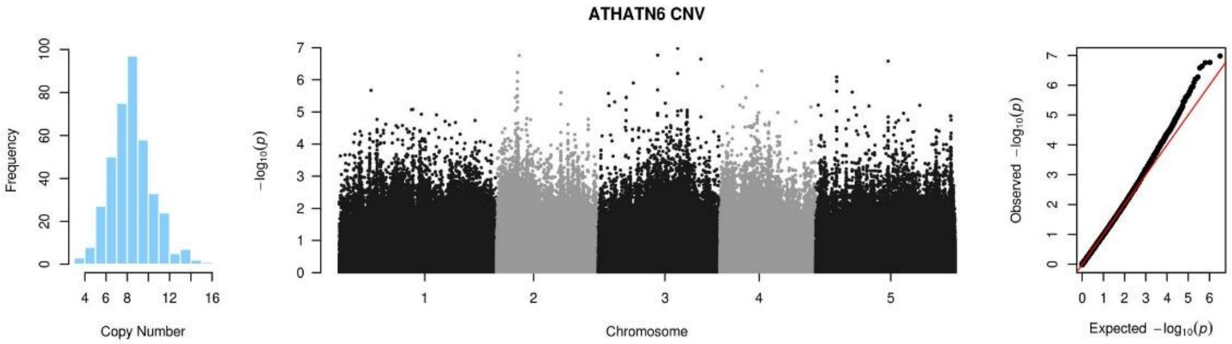

TE	GWAS associated interval	Leading SNP	Type of association	*P-value* Joined	Worldwide	Sweden	MAF	Candidate gene(s)

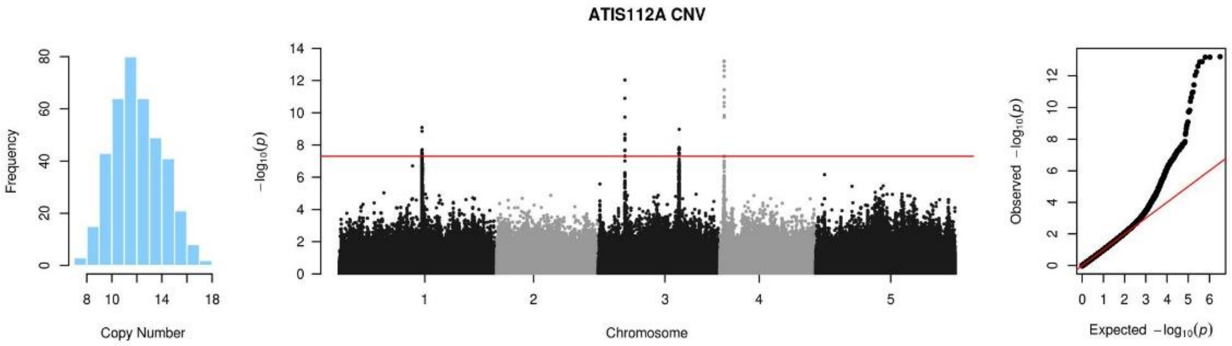

TE	GWAS associated interval	Leading SNP	Type of association	*P-value*			MAF	Candidate gene(s)
				Joined	Worldwide	Sweden		
ATIS112A	Chr1:15995694-16067310	Chr1-16031743	*CIS*	8.18E-10	8.97E-06	6.02E-06	0.336	
ATIS112A	Chr3:15617514-15642531	Chr3-5157268	*CIS*	9.19E-13	4.82E-08	1.41E-06	0.272	
ATIS112A	Chr3:5149758-5158701	Chr3-15642530	*CIS*	1.06E-09	1.70E-06	3.94E-04	0.436	
ATIS112A	Chr4:866297-871018	Chr4-866304	*CIS*	6.51E-14	3.51E-08	1.10E-06	0.369	

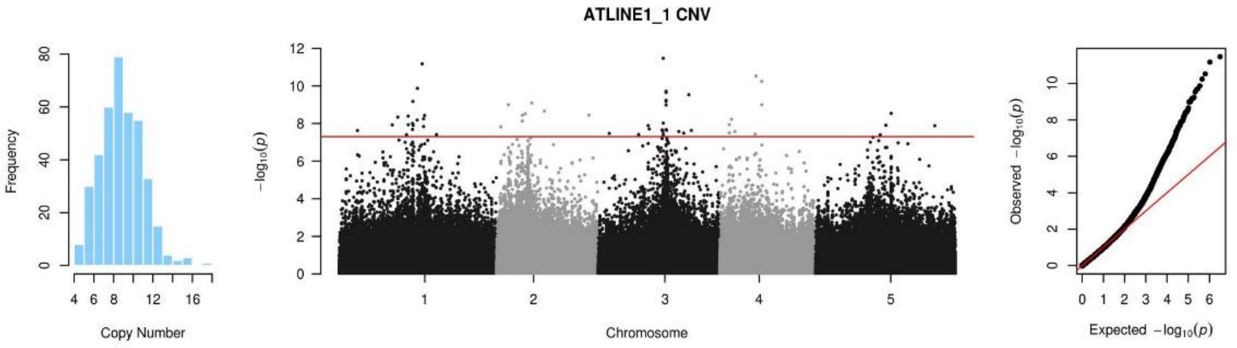

TE	GWAS associated interval	Leading SNP	Type of association	*P-value*			MAF	Candidate gene(s)
				Joined	Worldwide	Sweden		
ATLINE1_1	Chr1:3527270-3527272	Chr1-3527271	*TRANS*	2.35E-08	1.22E-05	5.89E-05	0.121	*AT1G10640, AT1G10650, AT1G10652, AT1G10657, AT1G10660, AT1G10670*
ATLINE1_1	Chr1:10280562-10280564	Chr1-10280563	*TRANS*	1.17E-08	2.06E-09	NA	0.256	*AT1G29350, AT1G29355, AT1G29357, AT1G29370, AT1G29380, AT1G29390, AT1G29395, AT1G29400*
ATLINE1_1	Chr1:11339021-11339023	Chr1-11339022	*TRANS*	4.56E-09	5.86E-07	NA	0.241	*AT1G31650, AT1G31660, AT1G31670, AT1G31690*
ATLINE1_1	Chr1:13354892-13365991	Chr1-13354893	*TRANS*	4.00E-09	1.33E-07	2.06E-03	0.441	*AT1G35900, AT1G35910*
ATLINE1_1	Chr1:14186622-14186631	Chr1-14186630	*TRANS*	9.81E-09	3.12E-10	1.34E-01	0.372	*AT1G37150, AT1G37162*
ATLINE1_1	Chr1:14263097-14313946	Chr1-14263098	*TRANS*	6.65E-10	1.95E-09	NA	0.244	*AT1G38065, AT1G38131*
ATLINE1_1	Chr1:15087421-16344119	Chr1-16073092	*TRANS*	6.68E-12	4.31E-10	NA	0.228	.
ATLINE1_1	Chr1:16518106-16518108	Chr1-16518107	*TRANS*	3.77E-09	1.02E-08	NA	0.397	*AT1G43730*
ATLINE1_1	Chr2:859602-859604	Chr2-859603	*TRANS*	1.49E-08	2.10E-05	2.80E-03	0.497	*AT2G02930, AT2G02950, AT2G02955, AT2G02960, AT2G02970, AT2G02980*
ATLINE1_1	Chr2:2304138-2304140	Chr2-2304139	*TRANS*	1.00E-09	5.01E-10	2.09E-01	0.374	*AT2G05970*
ATLINE1_1	Chr2:4999266-4999451	Chr2-4999267	*TRANS*	7.35E-09	6.20E-08	NA	0.105	*AT2G12400*
ATLINE1_1	Chr2:5558376-5558378	Chr2-5558377	*TRANS*	3.01E-09	1.30E-07	1.25E-02	0.362	*AT2G13370*
ATLINE1_1	Chr2:6822456-6822458	Chr2-6822457	*TRANS*	8.04E-10	1.88E-10	NA	0.328	*AT2G15620, AT2G15630, AT2G15640, AT2G15660, AT2G15670, AT2G15680, AT2G15690*
ATLINE1_1	Chr2:9281325-9281327	Chr2-9281326	*TRANS*	2.18E-09	8.82E-09	NA	0.182	*AT2G21710, AT2G21720, AT2G21725, AT2G21727, AT2G21730, AT2G21740, AT2G21750, AT2G21760, AT2G21770, AT2G21780*
ATLINE1_1	Chr2:17904473-17904475	Chr2-17904474	*TRANS*	3.57E-09	3.11E-07	2.18E-03	0.077	*AT2G43018, AT2G43020, AT2G43030, AT2G43040, AT2G43050, AT2G43060, AT2G43070*
ATLINE1_1	Chr3:9643029-9643031	Chr3-9643030	*TRANS*	1.29E-08	2.95E-08	4.83E-03	0.487	*AT3G26290, AT3G26300, AT3G26310, AT3G26320, AT3G26330, AT3G26340*
ATLINE1_1	Chr3:9859836-9859838	Chr3-9859837	*TRANS*	1.95E-08	1.98E-05	3.05E-05	0.474	*AT3G26780, AT3G26782, AT3G26790, AT3G26800, AT3G26810, AT3G26812*
ATLINE1_1	Chr3:12354058-12354060	Chr3-12354059	*TRANS*	2.18E-08	2.92E-08	6.09E-03	0.438	*AT3G30720, AT3G30725, AT3G30730*
ATLINE1_1	Chr3:12462649-12566676	Chr3-12536058	*TRANS*	3.37E-12	2.11E-10	3.02E-02	0.256	*AT3G30778, AT3G30805, AT3G30820*
ATLINE1_1	Chr3:13099797-13104190	Chr3-13099798	*CIS*	6.54E-10	1.71E-05	8.91E-06	0.395	
ATLINE1_1	Chr3:13246999-13247001	Chr3-13247000	*TRANS*	2.29E-08	3.75E-04	8.69E-06	0.341	*AT3G32280*
ATLINE1_1	Chr3:15588932-15588934	Chr3-15588933	*TRANS*	2.66E-08	1.11E-06	NA	0.151	*AT3G43682*
ATLINE1_1	Chr3:17519634-17519636	Chr3-17519635	*TRANS*	2.94E-10	5.92E-10	NA	0.269	*AT3G47510, AT3G47520, AT3G47530, AT3G47540, AT3G47543, AT3G47550, AT3G47560, AT3G47570*
ATLINE1_1	Chr3:17978865-17978867	Chr3-17978866	*TRANS*	2.33E-08	3.50E-07	NA	0.210	*AT3G48515, AT3G48520, AT3G48530*
ATLINE1_1	Chr4:1891430-1891432	Chr4-1891431	*TRANS*	1.15E-08	1.10E-08	4.33E-01	0.262	*AT4G03960, AT4G03965*
ATLINE1_1	Chr4:2298769-2298771	Chr4-2298770	*TRANS*	5.88E-09	7.50E-09	NA	0.310	*AT4G04570, AT4G04580*
ATLINE1_1	Chr4:2960759-2960761	Chr4-2960760	*TRANS*	2.66E-08	2.79E-08	NA	0.144	*AT4G05612*
ATLINE1_1	Chr4:7062242-7062244	Chr4-7062243	*TRANS*	2.97E-11	1.43E-11	NA	0.223	*AT4G11670, AT4G11680, AT4G11690, AT4G11700, AT4G11720, AT4G11730, AT4G11740*
ATLINE1_1	Chr4:8148250-8148313	Chr4-8148312	*TRANS*	5.72E-11	7.55E-11	NA	0.226	***AT4G14140 (MET2a)***
ATLINE1_1	Chr5:13603137-13603139	Chr5-13603138	*TRANS*	1.22E-08	4.04E-09	2.49E-02	0.387	*AT5G35375, AT5G35380, AT5G35390, AT5G35400, AT5G35405, AT5G35407*
ATLINE1_1	Chr5:14590846-14590848	Chr5-14590847	*TRANS*	2.86E-09	1.00E-07	1.76E-02	0.149	*AT5G36940, AT5G36950, AT5G36960, AT5G36940, AT5G36950, AT5G36960*
ATLINE1_1	Chr5:23050034-23050036	Chr5-23050035	*TRANS*	1.31E-08	1.68E-07	1.38E-03	0.292	*AT5G56960, AT5G56970, AT5G56975, AT5G56980*

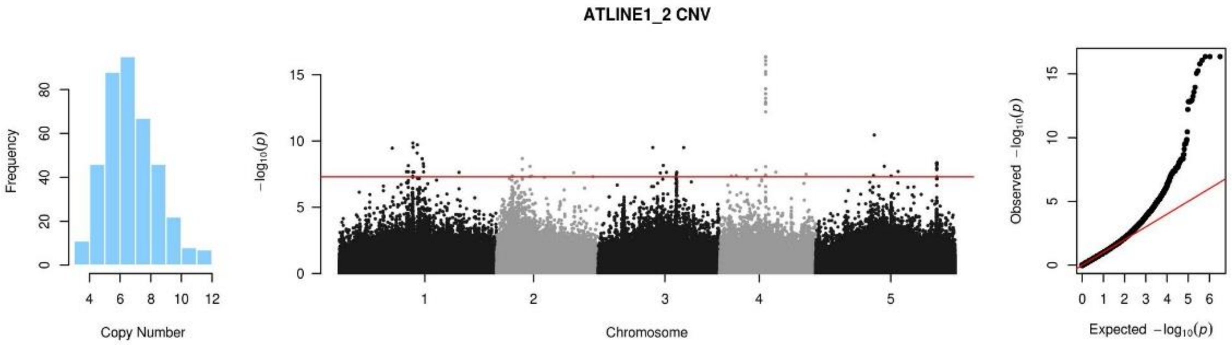

TE	GWAS associated interval	Leading SNP	Type of association	P-value			MAF	Candidate gene(s)
				Joined	Worldwide	Sweden		
ATLINE1_2	Chr1:10280562-10280564	Chr1-10280563	*TRANS*	3.50E-10	5.19E-11	NA	0.256	*AT1G29350, AT1G29355, AT1G29357, AT1G29370, AT1G29380, AT1G29390, AT1G29395, AT1G29400*
ATLINE1_2	Chr1:13013843-13013845	Chr1-13013844	*TRANS*	2.08E-08	3.80E-09	2.00E-01	0.297	*AT1G35375, AT1G35400*
ATLINE1_2	Chr1:13381001-13401451	Chr1-13381002	*TRANS*	7.29E-09	1.52E-09	NA	0.218	.
ATLINE1_2	Chr1:14225247-16344119	Chr1-14263098	*TRANS*	1.42E-10	1.05E-10	NA	0.244	.
ATLINE1_2	Chr1:23181917-23181919	Chr1-23181918	*TRANS*	2.30E-08	1.81E-06	4.37E-02	0.195	*AT1G62570, AT1G62580, AT1G62590, AT1G62600, AT1G62610, AT1G62620, AT1G62630*
ATLINE1_2	Chr2:4997864-4999451	Chr2-4999450	*TRANS*	2.10E-09	1.38E-11	NA	0.131	*AT2G12400*
ATLINE1_2	Chr2:6477378-6477380	Chr2-6477379	*TRANS*	8.13E-09	6.46E-11	2.74E-01	0.390	*AT2G15000, AT2G15010*
ATLINE1_2	Chr2:14932292-14932294	Chr2-14932293	*TRANS*	2.43E-08	6.27E-08	NA	0.392	*AT2G35520, AT2G35530, AT2G35540, AT2G35550, AT2G35580, AT2G35585, AT2G35600*
ATLINE1_2	Chr3:10542191-10542193	Chr3-10542192	*TRANS*	3.08E-10	2.25E-07	6.95E-05	0.441	*AT3G28243, AT3G28250, AT3G28260, AT3G28270, AT3G28280, AT3G28290, AT3G28291*
ATLINE1_2	Chr3:12001880-12001882	Chr3-12001881	*TRANS*	2.56E-08	7.67E-11	6.58E-01	0.233	*AT3G30387, AT3G30391*
ATLINE1_2	Chr3:12566674-12566676	Chr3-12566675	*TRANS*	6.91E-09	2.67E-09	NA	0.218	.
ATLINE1_2	Chr3:13199634-13199636	Chr3-13199635	*TRANS*	2.29E-08	9.21E-07	NA	0.315	.
ATLINE1_2	Chr3:15178231-15178298	Chr3-15178232	*CIS*	2.48E-08	6.54E-05	1.14E-04	0.090	
ATLINE1_2	Chr3:16511648-16511650	Chr3-16511649	*TRANS*	3.10E-10	1.07E-09	3.29E-02	0.200	*AT3G45100, AT3G45110, AT3G45130*
ATLINE1_2	Chr4:7062242-7062244	Chr4-7062243	*TRANS*	1.57E-08	8.76E-10	NA	0.223	*AT4G11670, AT4G11680, AT4G11690, AT4G11700, AT4G11720, AT4G11730, AT4G11740*
ATLINE1_2	Chr4:10898110-10898112	Chr4-10898111	*TRANS*	2.23E-08	6.07E-11	4.54E-02	0.249	*AT4G20140, AT4G20150, AT4G20160, AT4G20170, AT4G20190*
ATLINE1_2	Chr5:11344169-11344171	Chr5-11344170	*TRANS*	3.54E-11	2.05E-10	1.02E-02	0.408	.
ATLINE1_2	Chr5:13289118-13289120	Chr5-13289119	*TRANS*	8.00E-09	2.54E-06	NA	0.059	.
ATLINE1_2	Chr5:15962104-15962106	Chr5-15962105	*TRANS*	1.99E-08	2.96E-07	3.04E-02	0.156	*AT5G39860, AT5G39865, AT5G39870, AT5G39880*

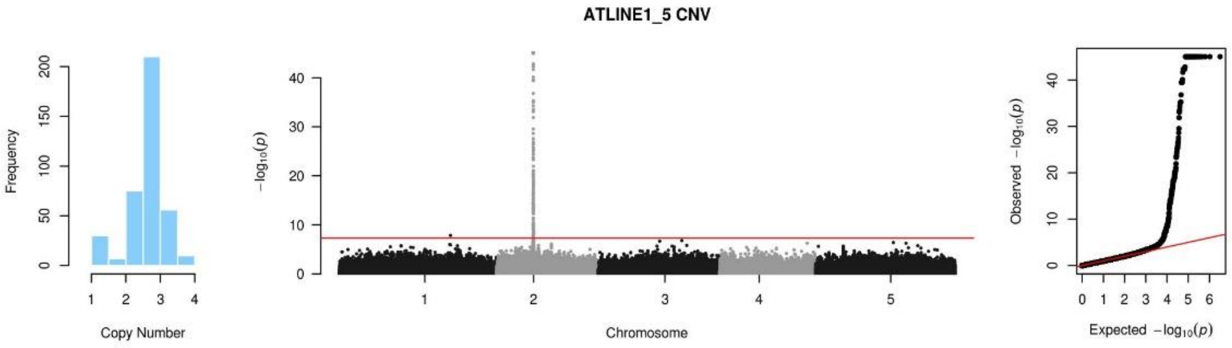

TE	GWAS associated interval	Leading SNP	Type of association	*P-value*			MAF	Candidate gene(s)
				Joined	Worldwide	Sweden		
ATLINE1_5	Chr1:21540447-21540449	Chr1-21540448	*TRANS*	1.35E-08	6.54E-05	8.67E-05	0.297	*AT1G58150, AT1G58160, AT1G58170, AT1G58180, AT1G58190, AT1G58200*
ATLINE1_5	Chr2:7057592-7215595	Chr2-7106640	*CIS*	8.97E-46	1.84E-35	NA	0.082	

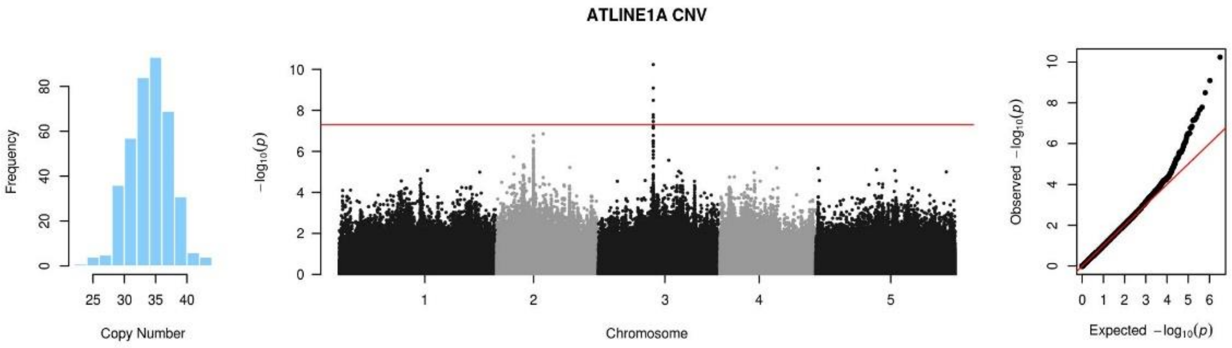

TE	GWAS associated interval	Leading SNP	Type of association	*P-value*			MAF	Candidate gene(s)
				Joined	Worldwide	Sweden		
ATLINE1A	Chr3:10642015-10645602	Chr3-10642086	*CIS*	5.84E-11	3.18E-08	4.47E-04	0.459	

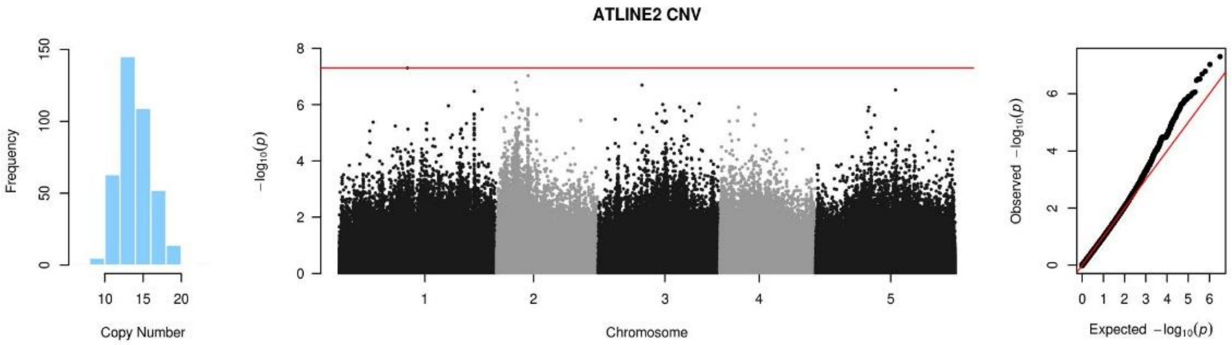

TE	GWAS associated interval	Leading SNP	Type of association	*P-value*			MAF	Candidate gene(s)
				Joined	Worldwide	Sweden		

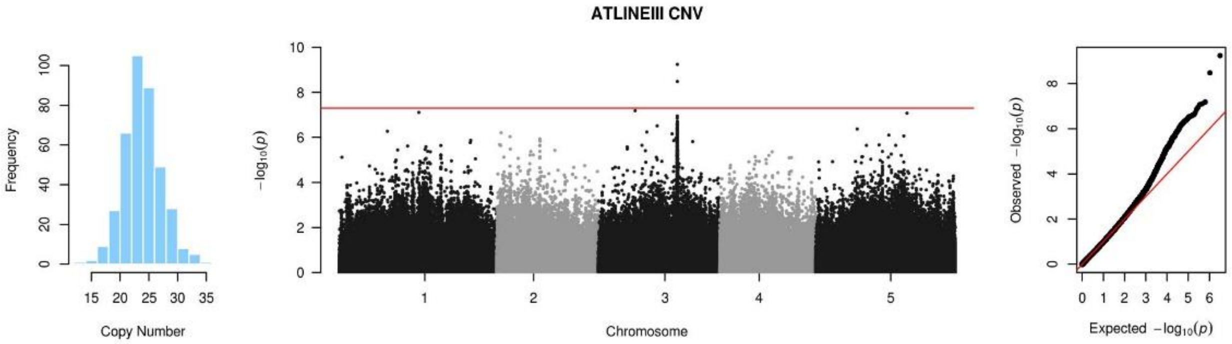

TE	GWAS associated interval	Leading SNP	Type of association	*P-value*			MAF	Candidate gene(s)
				Joined	Worldwide	Sweden		
ATLINEIII	Chr3:15295284-15295304	Chr3-15295303	*TRANS*	5.73E-10	9.29E-07	1.25E-04	0.282	*AT3G43340*

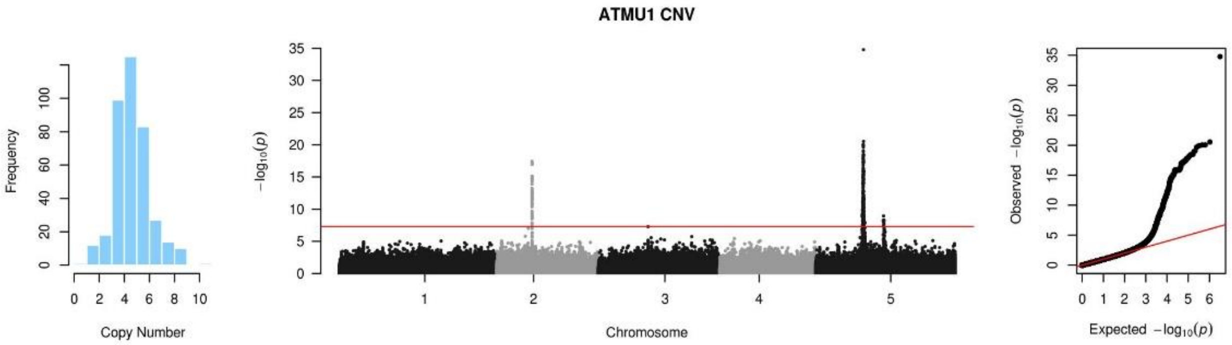

TE	GWAS associated interval	Leading SNP	Type of association	*P-value*			MAF	Candidate gene(s)
				Joined	Worldwide	Sweden		
ATMU1	Chr2:6874717-6891586	Chr2-6882611	*CIS*	3.75E-18	4.16E-13	4.94E-05	0.100	
ATMU1	Chr5:9047986-9522014	Chr5-9260607	*CIS*	1.67E-35	3.85E-20	8.24E-18	0.105	
ATMU1	Chr5:13113294-13230580	Chr5-13113295	*TRANS*	5.78E-09	9.67E-08	NA	0.051	*AT5G34850, AT5G34870, AT5G34881, AT5G34882, AT5G34883, AT5G34885, AT5G34887, AT5G34905, AT5G34908, AT5G34930, AT5G34940*

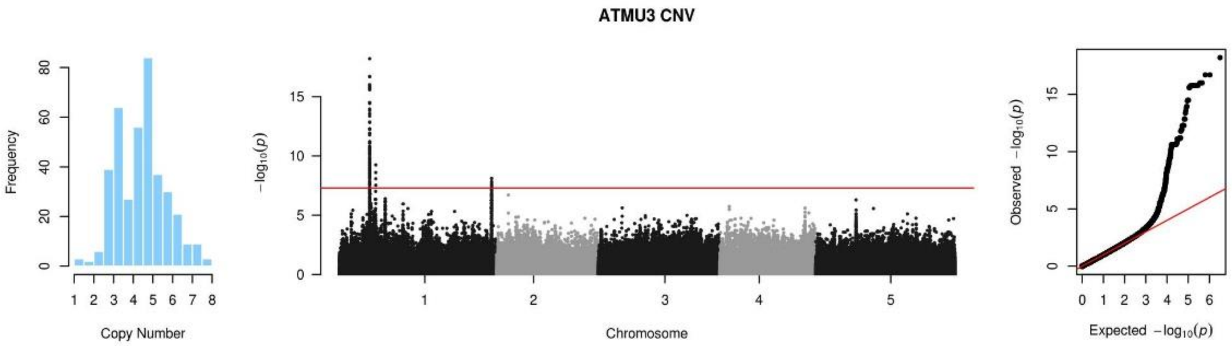

TE	GWAS associated interval	Leading SNP	Type of association	*P-value*			MAF	Candidate gene(s)
				Joined	Worldwide	Sweden		
ATMU3	Chr1:7082827-7084454	Chr1-5905999	*CIS*	6.17E-19	3.21E-06	4.68E-16	0.195	
ATMU3	Chr1:29472077-29482991	Chr1-7082828	*TRANS*	5.59E-10	3.90E-07	NA	0.100	*AT1G20410*
ATMU3	Chr1:5876536-5922541	Chr1-29472078	*CIS*	7.91E-09	5.16E-06	1.31E-03	0.128	

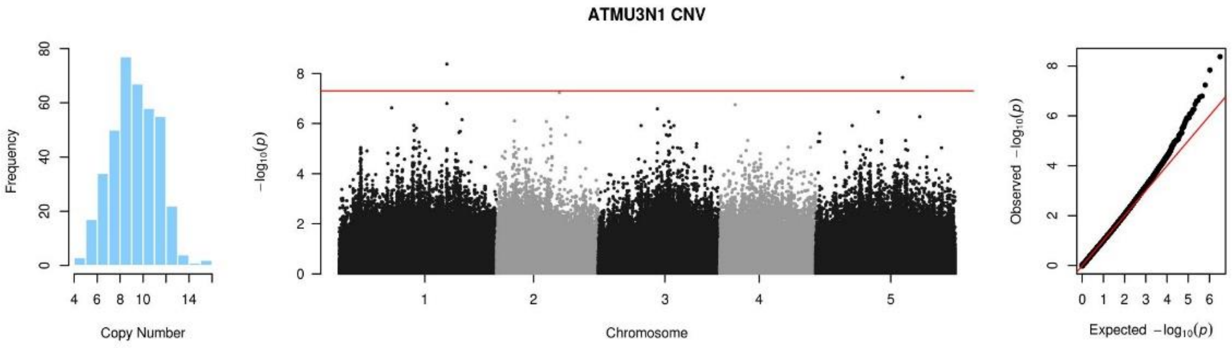

TE	GWAS associated interval	Leading SNP	Type of association	*P-value* Joined	Worldwide	Sweden	MAF	Candidate gene(s)
ATMU3N1	Chr1:20822319-20822321	Chr1-20822320	*TRANS*	4.24E-09	NA	4.27E-06	0.382	*AT1G55690, AT1G55700, AT1G55710, AT1G55720, AT1G55730*
ATMU3N1	Chr5:16828813-16828815	Chr5-16828814	*TRANS*	1.45E-08	NA	6.87E-06	0.177	*AT5G42060, AT5G42070, AT5G42080, AT5G42090, AT5G42092, AT5G42100, AT5G42110, AT5G42120, AT5G42130, AT5G42140*

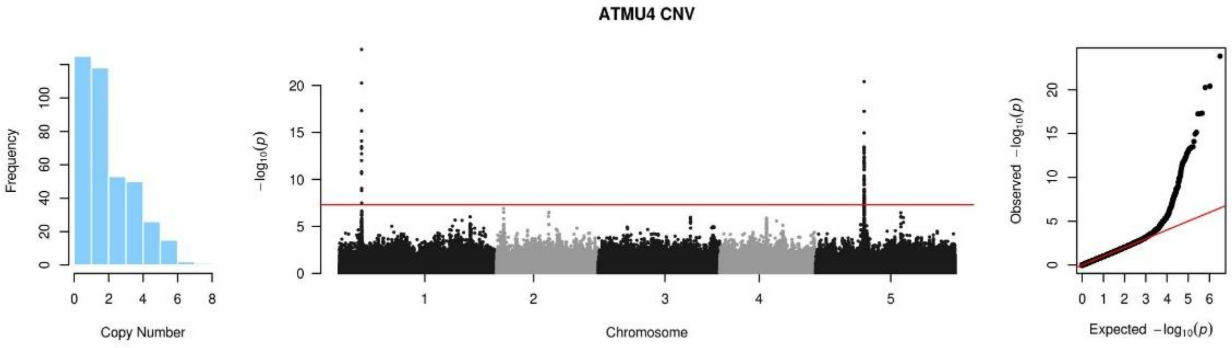

TE	GWAS associated interval	Leading SNP	Type of association	*P-value* Joined	Worldwide	Sweden	MAF	Candidate gene(s)
ATMU4	Chr1:4330246-4336252	Chr1-4332459	*CIS*	1.50E-24	4.70E-18	1.16E-08	0.423	
ATMU4	Chr5:9359695-9398879	Chr5-9374600	*CIS*	3.90E-21	1.17E-13	1.02E-09	0.233	

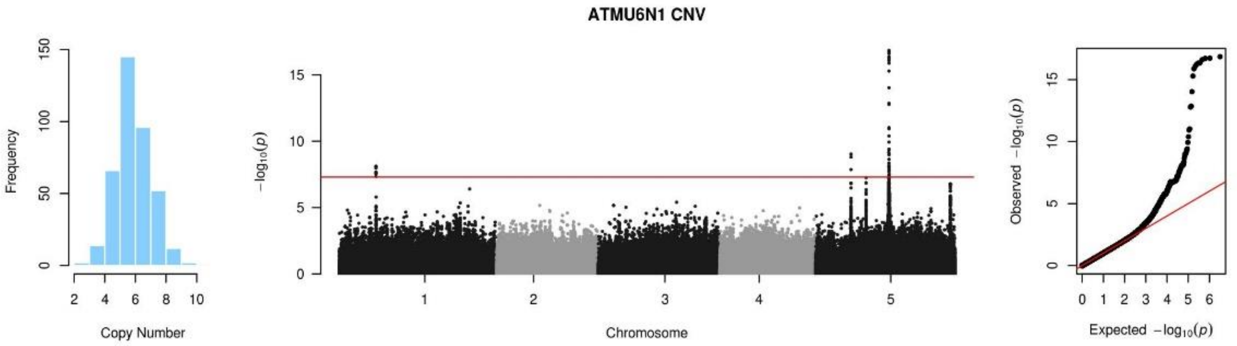

TE	GWAS associated interval	Leading SNP	Type of association	*P-value* Joined	Worldwide	Sweden	MAF	Candidate gene(s)
ATMU6N1	Chr1:7112320-7126770	Chr1-7121312	*CIS*	7.92E-09	3.10E-06	4.33E-04	0.051	
ATMU6N1	Chr5:6837888-6840327	Chr5-6837889	*CIS*	9.27E-10	1.16E-08	8.46E-04	0.308	
ATMU6N1	Chr5:14156862-14241288	Chr5-14196547	*CIS*	4.54E-17	1.63E-09	1.14E-09	0.362	

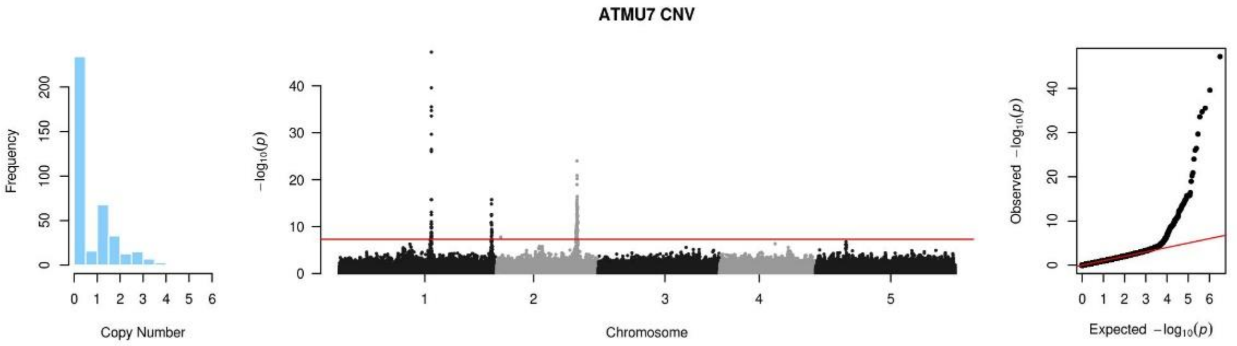

TE	GWAS associated interval	Leading SNP	Type of association	*P-value* Joined	Worldwide	Sweden	MAF	Candidate gene(s)
ATMU7	Chr1:17827343-17850172	Chr1-17843917	*CIS*	6.51E-48	1.04E-30	7.78E-20	0.195	
ATMU7	Chr1:29606534-29606536	Chr1-29606535	*TRANS*	1.49E-09	NA	1.45E-04	0.059	*AT1G78690, AT1G78700, AT1G78710, AT1G78720, AT1G78730, AT1G78740, AT1G78750, AT1G78760*
ATMU7	Chr2:853711-853718	Chr2-853712	*CIS*	1.84E-08	9.63E-06	6.79E-04	0.062	
ATMU7	Chr2:15400354-15688272	Chr2-15595243	*CIS*	1.02E-10	NA	8.13E-07	0.115	

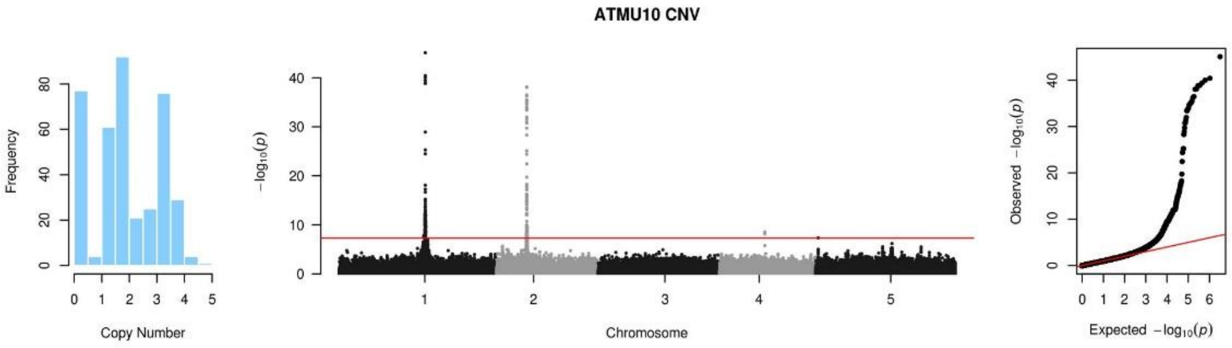

TE	GWAS associated interval	Leading SNP	Type of association	*P-value* Joined	Worldwide	Sweden	MAF	Candidate gene(s)
ATMU10	Chr1:16647700-16712103	Chr1-16660193	*CIS*	8.04E-46	2.21E-24	1.35E-24	0.390	
ATMU10	Chr2:5835416-5914331	Chr2-5861365	*CIS*	8.62E-39	4.42E-21	1.17E-18	0.441	
ATMU10	Chr4:8780515-8780544	Chr4-8780516	*TRANS*	2.81E-09	5.31E-05	7.00E-05	0.200	*AT4G15360, AT4G15370, AT4G15380*

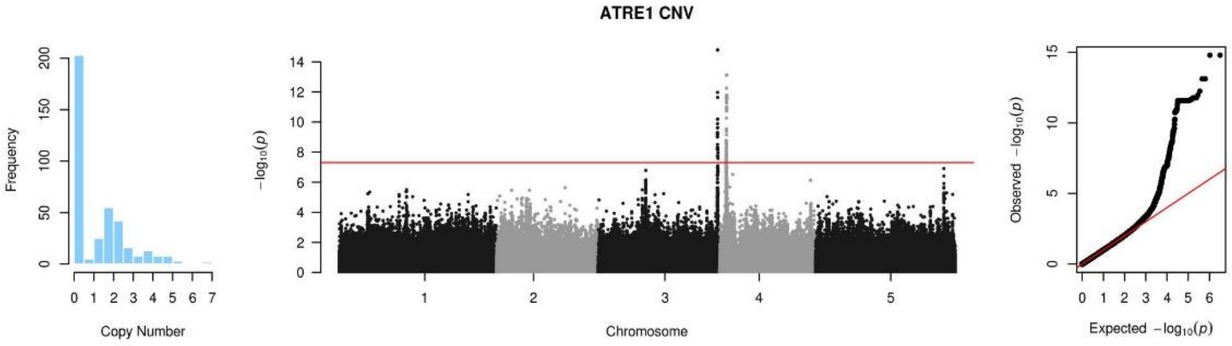

TE	GWAS associated interval	Leading SNP	Type of association	*P-value* Joined	Worldwide	Sweden	MAF	Candidate gene(s)
ATRE1	Chr3:23050888-23163122	Chr3-23079848	*TRANS*	1.61E-15	2.17E-11	1.04E-05	0.121	Telomere
ATRE1	Chr4:1259349-1373555	Chr4-1363394	*TRANS*	7.59E-14	5.83E-12	1.70E-04	0.182	Telomere

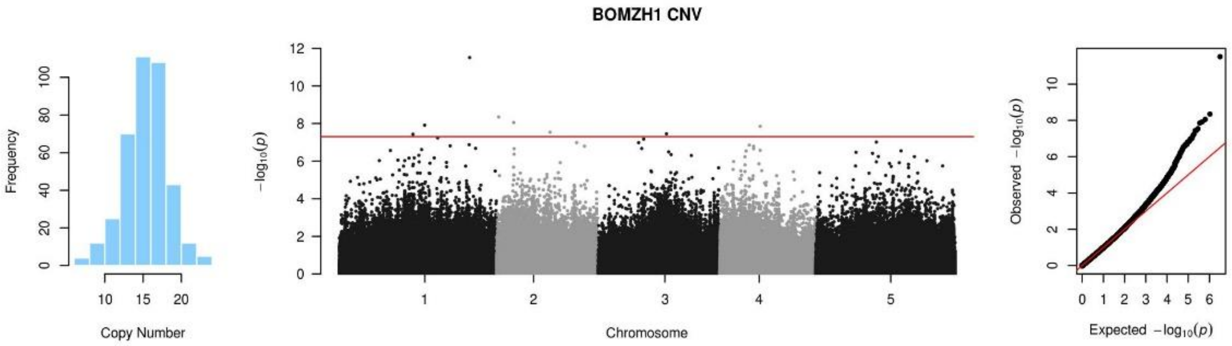

TE	GWAS associated interval	Leading SNP	Type of association	*P-value*			MAF	Candidate gene(s)
				Joined	Worldwide	Sweden		
BOMZH1	Chr1:16538277-16538279	Chr1-16538278	*TRANS*	1.20E-08	NA	1.99E-07	0.077	*AT1G43760, AT1G43765*
BOMZH1	Chr1:25245332-25245334	Chr1-25245333	*TRANS*	3.10E-12	NA	2.00E-09	0.136	*AT1G67390*
BOMZH1	Chr2:425001-425003	Chr2-425002	*TRANS*	4.49E-09	NA	1.88E-06	0.056	*AT2G01905, AT2G01910, AT2G01913, AT2G01918, AT2G01920, AT2G01930, AT2G01940*
BOMZH1	Chr2:3336992-3336994	Chr2-3336993	*TRANS*	8.84E-09	NA	NA	0.449	*AT2G07689, AT2G07691, AT2G07692, AT2G07695, AT2G07755, AT2G07792*
BOMZH1	Chr2:10367486-10367488	Chr2-10367487	*TRANS*	2.87E-08	NA	3.57E-06	0.105	*AT2G24340, AT2G24350, AT2G24360, AT2G24370, AT2G24380, AT2G24390, AT2G24395*
BOMZH1	Chr4:7861176-7861178	Chr4-7861177	*TRANS*	1.39E-08	NA	6.15E-06	0.054	*AT4G13500, AT4G13505, AT4G13510, AT4G13520, AT4G13530, AT4G13540, AT4G13550*

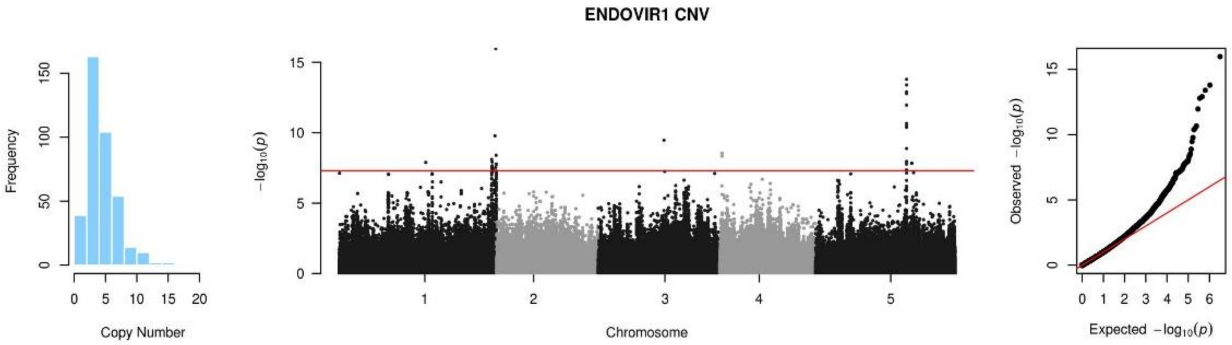

TE	GWAS associated interval	Leading SNP	Type of association	*P-value*			MAF	Candidate gene(s)
				Joined	Worldwide	Sweden		
ENDOVIR1	Chr1:16742503-16742505	Chr1-16742504	*TRANS*	1.27E-08	NA	7.46E-09	0.105	*AT1G44050*
ENDOVIR1	Chr1:29491625-29610696	Chr1-29491626	*TRANS*	7.81E-09	NA	1.00E-07	0.054	Centromere
ENDOVIR1	Chr1:30145534-30424738	Chr1-30145535	*TRANS*	1.67E-10	NA	4.98E-10	0.105	Centromere
ENDOVIR1	Chr3:12711190-12711192	Chr3-12711191	*TRANS*	3.39E-10	NA	2.07E-07	0.087	*AT3G31350*
ENDOVIR1	Chr4:482154-489967	Chr4-482155	*TRANS*	4.60E-09	NA	2.54E-06	0.092	*AT4G01090, AT4G01100, AT4G01110, AT4G01120, AT4G01130, AT4G01140, AT4G01150, AT4G01160, AT4G01170*
ENDOVIR1	Chr5:17598754-17603687	Chr5-17603169	*CIS*	4.02E-14	1.16E-10	7.69E-07	0.349	
ENDOVIR1	Chr5:18585594-18591343	Chr5-18585595	*TRANS*	1.46E-08	NA	1.94E-05	0.059	*AT5G45800, AT5G45810, AT5G45820, AT5G45830, AT5G45840, AT5G45850*

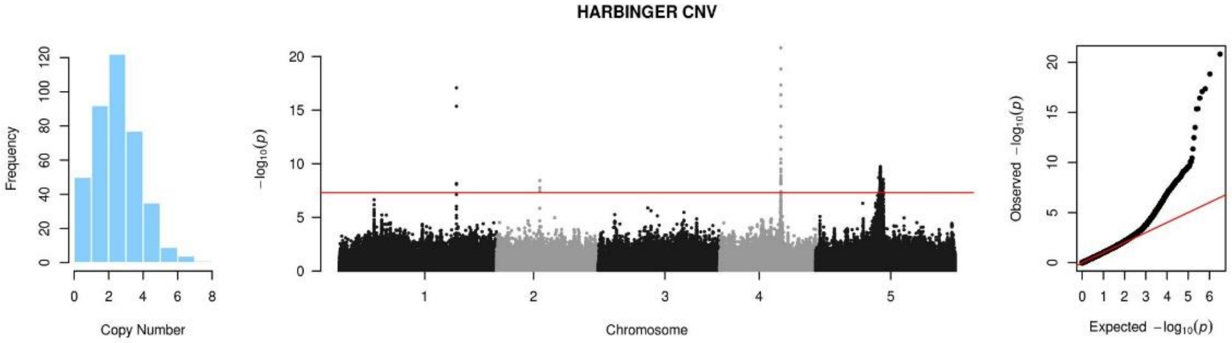

TE	GWAS associated interval	Leading SNP	Type of association	*P-value* Joined	Worldwide	Sweden	MAF	Candidate gene(s)
HARBINGER	Chr1:22695813-22698956	Chr1-22698955	*CIS*	6.77E-09	NA	3.33E-06	0.362	
HARBINGER	Chr2:8379751-8381366	Chr2-8381365	*CIS*	3.67E-09	4.17E-06	6.75E-04	0.482	
HARBINGER	Chr4:11828799-11840680	Chr4-11832740	*CIS*	1.54E-21	3.86E-09	7.78E-14	0.462	
HARBINGER	Chr5:12192380-13075605	Chr5-12443314	*CIS*	6.79E-10	2.25E-09	4.97E-04	0.274	

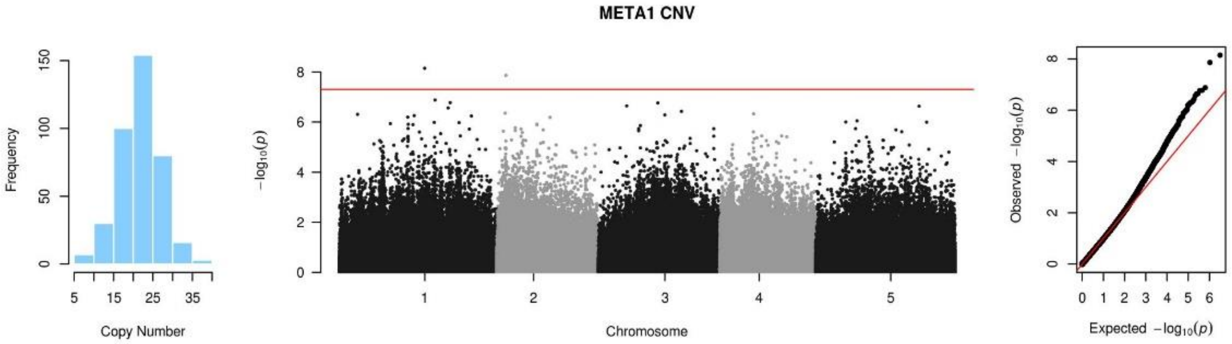

TE	GWAS associated interval	Leading SNP	Type of association	*P-value* Joined	Worldwide	Sweden	MAF	Candidate gene(s)
META1	Chr1:16538277-16538279	Chr1-16538278	*TRANS*	7.15E-09	NA	5.94E-09	0.156	*AT1G43765*
META1	Chr2:1825193-1825195	Chr2-1825194	*CIS*	1.38E-08	4.24E-04	4.02E-07	0.238	

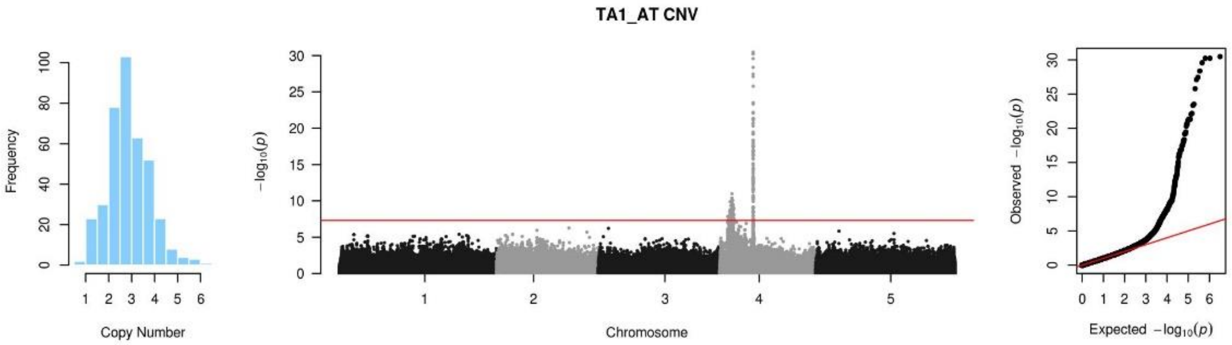

TE	GWAS associated interval	Leading SNP	Type of association	*P-value*			MAF	Candidate gene(s)
				Joined	Worldwide	Sweden		
TA1_AT	Chr4:1603072-2778767	Chr4-2405296	*TRANS*	3.97E-11	2.16E-09	NA	0.105	Centromere
TA1_AT	Chr4:6501683-6522039	Chr4-6502694	*CIS*	3.38E-31	1.46E-21	3.64E-13	0.315	

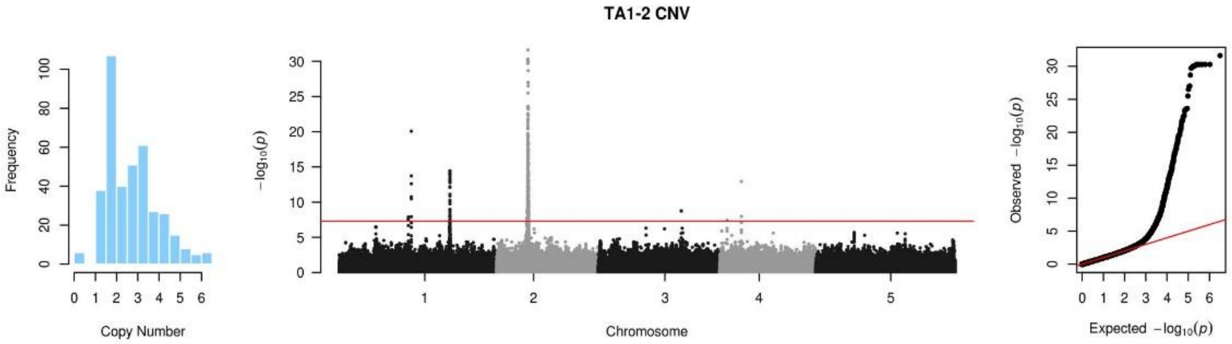

TE	GWAS associated interval	Leading SNP	Type of association	*P-value*			MAF	Candidate gene(s)
				Joined	Worldwide	Sweden		
TA1-2	Chr1:13374338-13959108	Chr1-13959107	*CIS*	1.17E-08	3.46E-01	1.22E-10	0.500	
TA1-2	Chr1:21421680-21427870	Chr1-21426003	*CIS*	1.78E-10	2.82E-03	3.51E-11	0.331	
TA1-2	Chr2:6047365-6202913	Chr2-6098822	*TRANS*	2.41E-32	1.80E-15	1.08E-17	0.277	*AT2G14255, AT2G14260, AT2G14270, AT2G14282, AT2G14285, AT2G14288, AT2G14289, AT2G14290, AT2G14365, AT2G14378, AT2G14390, AT2G14440, AT2G14460, AT2G14500, AT2G14510, AT2G14520, AT2G14530, AT2G14540*
TA1-2	Chr3:16053230-16053253	Chr3-16053231	*TRANS*	1.78E-09	NA	4.23E-08	0.064	*AT3G44400*
TA1-2	Chr4:4231227-4231267	Chr4-4231228	*TRANS*	1.15E-13	8.77E-12	2.25E-04	0.144	Centromere

TAG2 CNV

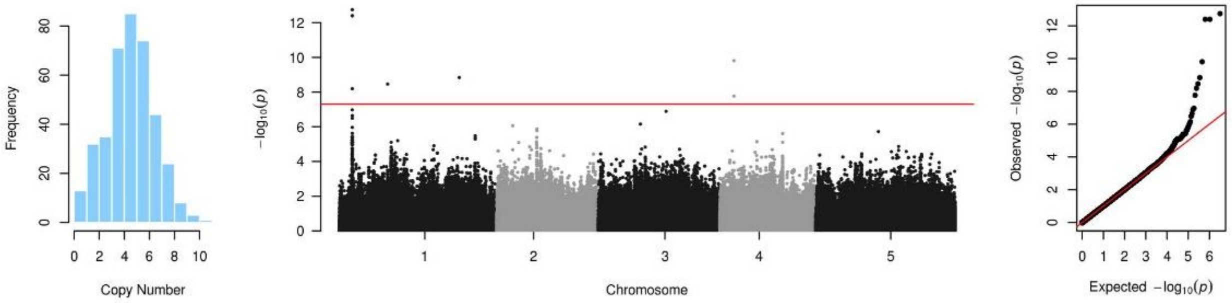

TE	GWAS associated interval	Leading SNP	Type of association	*P-value*			MAF	Candidate gene(s)
				Joined	Worldwide	Sweden		
TAG2	Chr1:2540827-2546070	Chr1-2540828	*TRANS*	1.81E-13	8.71E-11	7.25E-05	0.423	*AT1G08130*
TAG2	Chr1:9386433-9386435	Chr1-9386434	*TRANS*	3.47E-09	6.03E-04	5.61E-06	0.162	*AT1G27000, AT1G27008, AT1G27020, AT1G27030, AT1G27040, AT1G27045, AT1G27050, AT1G27060*
TAG2	Chr1:23242799-23242801	Chr1-23242800	*CIS*	1.44E-09	NA	2.67E-10	0.254	
TAG2	Chr4:2790842-2791555	Chr4-2790843	*CIS*	1.56E-10	4.06E-07	3.63E-04	0.490	

TSCL CNV

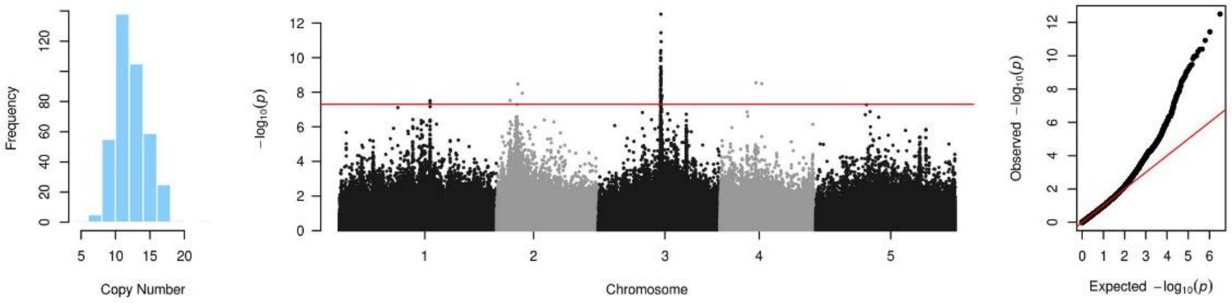

TE	GWAS associated interval	Leading SNP	Type of association	*P-value*			MAF	Candidate gene(s)
				Joined	Worldwide	Sweden		
TSCL	Chr1:17576803-17576805	Chr1-17576804	*TRANS*	3.08E-08	1.54E-05	1.12E-03	0.438	*AT1G47740, AT1G47750, AT1G47760, AT1G47765, AT1G47740, AT1G47750, AT1G47760, AT1G47765*
TSCL	Chr2:2644617-2644619	Chr2-2644618	*TRANS*	2.99E-08	2.01E-07	1.33E-02	0.500	*AT2G06645*
TSCL	Chr2:4129746-4129748	Chr2-4129747	*TRANS*	3.37E-09	1.91E-07	1.95E-02	0.382	*AT2G10602*
TSCL	Chr2:4997864-4997866	Chr2-4997865	*TRANS*	1.13E-08	2.32E-09	NA	0.241	*AT2G12400*
TSCL	Chr3:12042050-12343556	Chr3-12117064	*CIS*	3.11E-13	1.58E-08	4.66E-07	0.387	
TSCL	Chr4:7062242-7062244	Chr4-7062243	*TRANS*	2.85E-09	2.45E-09	NA	0.223	*AT4G11670, AT4G11680, AT4G11690, AT4G11700, AT4G11720, AT4G11730, AT4G11740*
TSCL	Chr4:8148311-8148313	Chr4-8148312	*TRANS*	3.19E-09	1.21E-09	NA	0.226	***AT4G14140 (MET2a)***

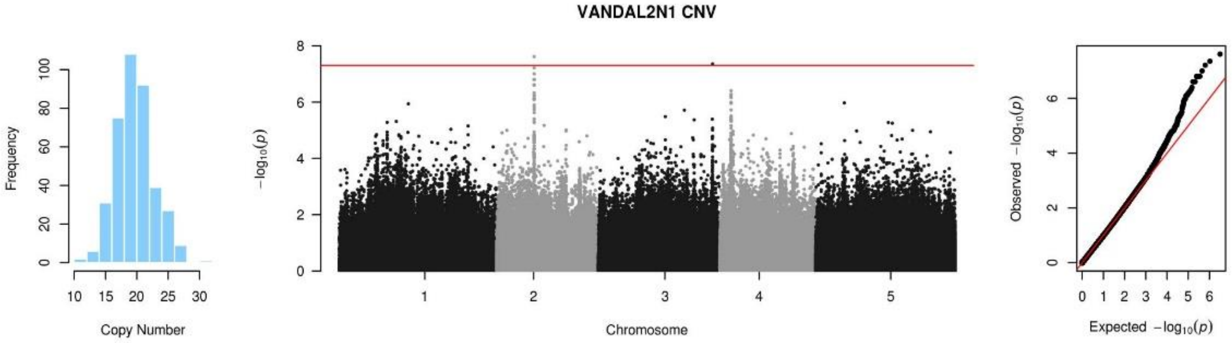

TE	GWAS associated interval	Leading SNP	Type of association	*P-value* Joined	Worldwide	Sweden	MAF	Candidate gene(s)
VANDAL2N1	Chr2:7287475-7287477	Chr2-7287476	*CIS*	2.42E-08	1.66E-06	2.72E-03	0.100	

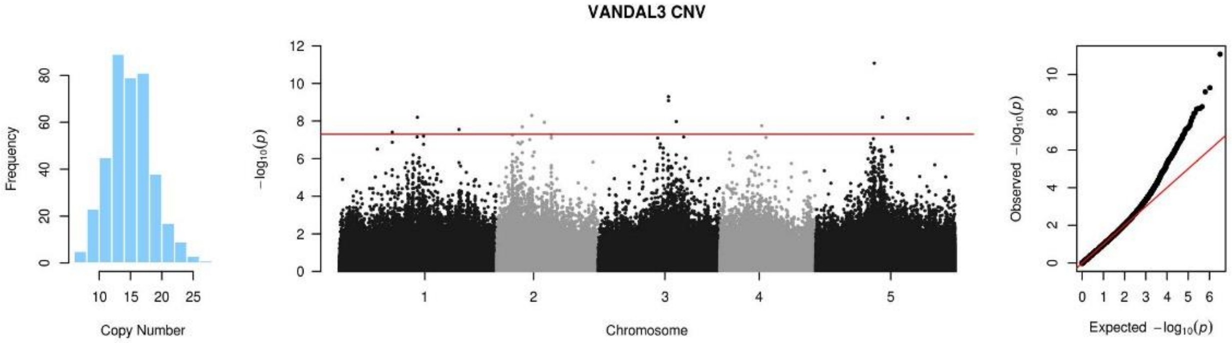

TE	GWAS associated interval	Leading SNP	Type of association	*P-value* Joined	Worldwide	Sweden	MAF	Candidate gene(s)
VANDAL3	Chr1:15141763-15141765	Chr1-15141764	*TRANS*	6.41E-09	1.42E-07	NA	0.136	*AT1G62570, AT1G62580, AT1G62590, AT1G62600, AT1G62610, AT1G62620, AT1G62630*
VANDAL3	Chr1:23179645-23179647	Chr1-23179646	*TRANS*	2.84E-08	5.69E-07	5.28E-02	0.328	*AT1G62570, AT1G62580, AT1G62590, AT1G62600, AT1G62610, AT1G62620, AT1G62630*
VANDAL3	Chr2:4999449-4999451	Chr2-4999450	*TRANS*	2.02E-08	3.40E-08	NA	0.131	*AT2G12400*
VANDAL3	Chr2:6822456-6822458	Chr2-6822457	*TRANS*	5.07E-09	1.71E-08	NA	0.328	*AT2G15620, AT2G15630, AT2G15640, AT2G15660, AT2G15670, AT2G15680, AT2G15690*
VANDAL3	Chr2:9281325-9281327	Chr2-9281326	*TRANS*	1.16E-08	9.39E-08	NA	0.182	*AT2G21710, AT2G21720, AT2G21725, AT2G21727, AT2G21730, AT2G21740, AT2G21750, AT2G21760, AT2G21770, AT2G21780*
VANDAL3	Chr3:13582231-13582240	Chr3-13582232	*TRANS*	8.30E-10	7.51E-09	NA	0.141	Centromere
VANDAL3	Chr3:15074728-15074730	Chr3-15074729	*TRANS*	1.06E-08	9.15E-08	NA	0.167	*AT3G43083*
VANDAL3	Chr4:8148311-8148313	Chr4-8148312	*TRANS*	1.76E-08	1.90E-07	NA	0.226	***AT4G14140 (MET2a)***
VANDAL3	Chr5:11344169-11344171	Chr5-11344170	*TRANS*	8.34E-12	3.65E-12	1.77E-01	0.408	Centromere
VANDAL3	Chr5:12931127-12931129	Chr5-12931128	*TRANS*	6.34E-09	4.50E-07	1.65E-03	0.362	Centromere
VANDAL3	Chr5:17851103-17851105	Chr5-17851104	*TRANS*	7.04E-09	2.03E-08	1.25E-01	0.192	*AT5G44290, AT5G44300, AT5G44306, AT5G44310, AT5G44316, AT5G44320, AT5G44330, AT5G44340*

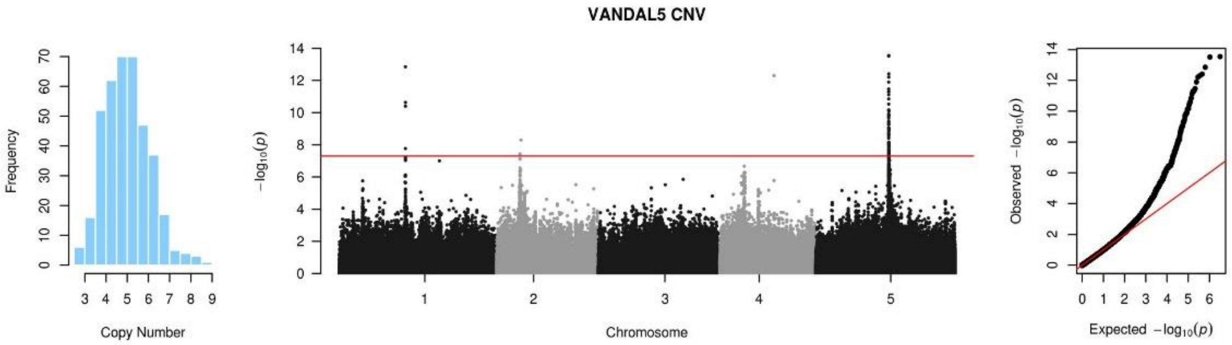

TE	GWAS associated interval	Leading SNP	Type of association	*P-value* Joined	Worldwide	Sweden	MAF	Candidate gene(s)
VANDAL5	Chr1:12818394-12828989	Chr1-12818395	*CIS*	1.40E-13	2.62E-08	7.05E-07	0.431	
VANDAL5	Chr2:4737737-4737739	Chr2-4737738	*TRANS*	5.02E-09	3.31E-07	4.82E-03	0.118	*AT2G11778, AT2G11810*
VANDAL5	Chr4:10536648-10536650	Chr4-10536649	*CIS*	4.94E-13	2.47E-10	1.50E-04	0.287	
VANDAL5	Chr5:14113255-14113257	Chr5-14113256	*TRANS*	7.55E-09	3.58E-04	9.64E-08	0.462	*AT5G35950, AT5G35960, AT5G35970*

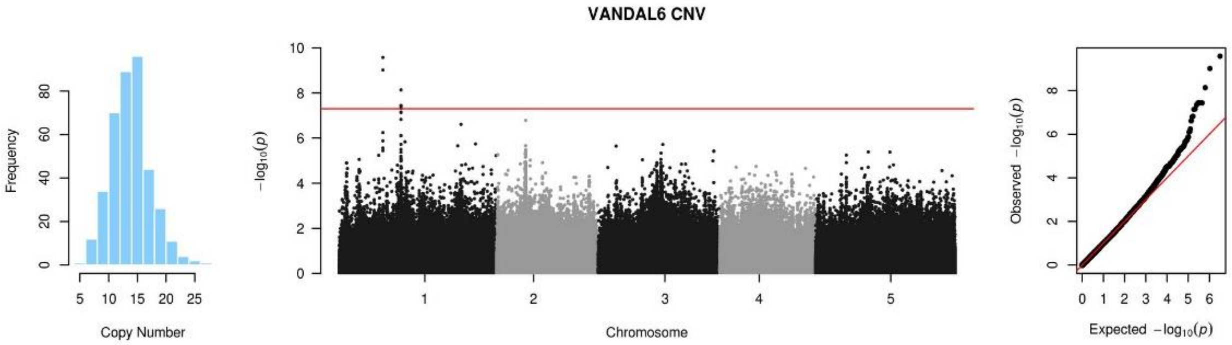

TE	GWAS associated interval	Leading SNP	Type of association	*P-value* Joined	Worldwide	Sweden	MAF	Candidate gene(s)
VANDAL6	Chr1:8461393-8461722	Chr1-8461394	*CIS*	9.70E-10	4.17E-05	1.46E-06	0.418	
VANDAL6	Chr1:11960663-11960665	Chr1-11960664	*TRANS*	7.33E-09	1.11E-03	2.52E-06	0.282	*AT1G32970, AT1G32975, AT1G32980, AT1G32990, AT1G32992, AT1G33010, AT1G33020, AT1G33030, AT1G33040, AT1G33050*

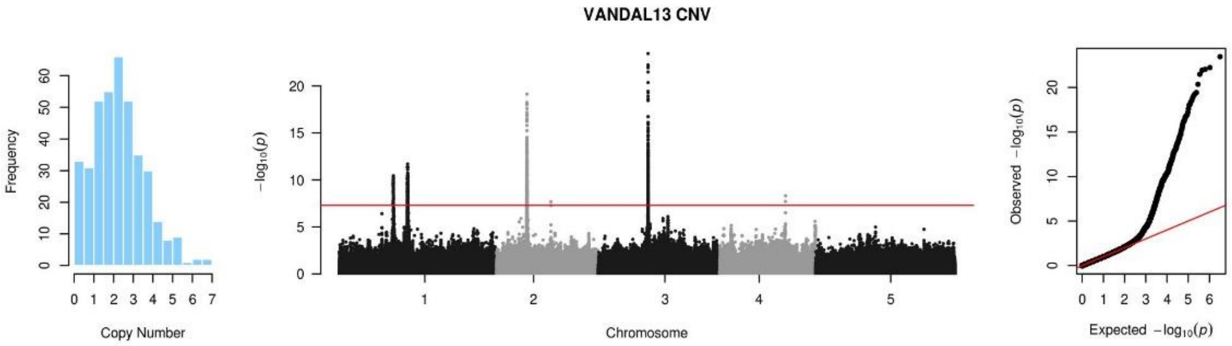

TE	GWAS associated interval	Leading SNP	Type of association	*P-value*			MAF	Candidate gene(s)
				Joined	Worldwide	Sweden		
VANDAL13	Chr1:10425512-10511828	Chr1-10452544	*CIS*	1.00E-10	2.01E-03	1.72E-09	0.321	
VANDAL13	Chr1:13229289-13278414	Chr1-13229290	*CIS*	4.71E-12	1.68E-07	2.65E-07	0.195	
VANDAL13	Chr2:5875322-5908135	Chr2-5903083	*CIS*	7.50E-20	9.12E-11	1.13E-10	0.464	
VANDAL13	Chr2:10530058-10530060	Chr2-10530059	*TRANS*	2.09E-08	4.82E-05	9.67E-05	0.474	*AT2G24740*
VANDAL13	Chr3:9591516-9634713	Chr3-9615484	*CIS*	3.54E-24	2.39E-13	5.30E-12	0.472	
VANDAL13	Chr4:12727871-12749741	Chr4-12749740	*TRANS*	4.86E-09	NA	3.50E-06	0.067	*AT4G24640, AT4G24644, AT4G24650, AT4G24660, AT4G24670, AT4G24680, AT4G24690, AT4G24700, AT4G24710, AT4G24730, AT4G24740, AT4G24750*

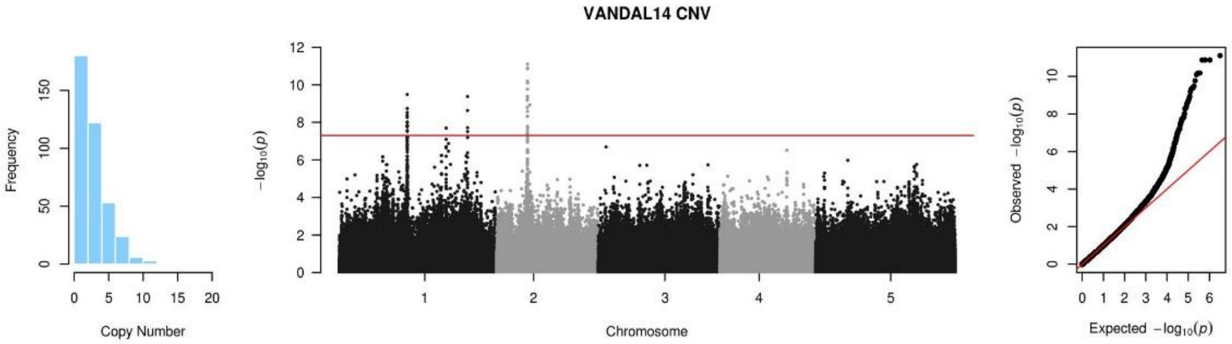

TE	GWAS associated interval	Leading SNP	Type of association	*P-value*			MAF	Candidate gene(s)
				Joined	Worldwide	Sweden		
VANDAL14	Chr1:20700191-20700193	Chr1-20700192	*TRANS*	2.00E-08	7.44E-02	3.32E-08	0.074	*AT1G55420, AT1G55430, AT1G55440, AT1G55450, AT1G55460*
VANDAL14	Chr1:24822615-24824216	Chr1-24823448	*TRANS*	4.22E-10	NA	5.65E-07	0.141	*AT1G66550*
VANDAL14	Chr2:5953772-6016808	Chr2-6014486	*CIS*	7.81E-12	3.61E-08	6.49E-06	0.249	
VANDAL14	Chr2:6449392-6449394	Chr2-6449393	*TRANS*	1.16E-09	1.14E-06	7.63E-05	0.085	*AT2G14960*

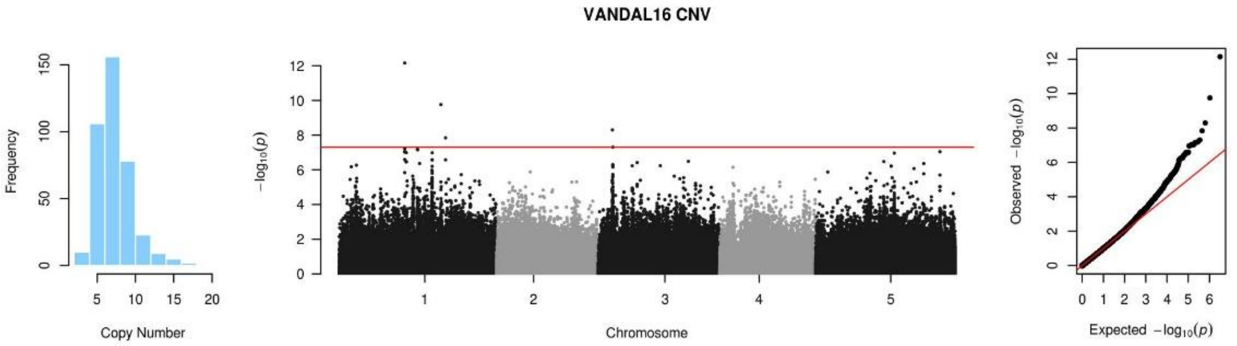

TE	GWAS associated interval	Leading SNP	Type of association	*P-value* Joined	Worldwide	Sweden	MAF	Candidate gene(s)
VANDAL16	Chr1:12678935-12678937	Chr1-12678936	*CIS*	7.03E-13	4.66E-08	9.88E-06	0.438	
VANDAL16	Chr1:19668640-19668642	Chr1-19668641	*TRANS*	1.75E-10	NA	1.35E-06	0.051	*AT1G52780, AT1G52790, AT1G52800, AT1G52810, AT1G52820, AT1G52825, AT1G52827, AT1G52830*
VANDAL16	Chr1:20580746-20580748	Chr1-20580747	*TRANS*	1.44E-08	6.56E-09	2.11E-01	0.090	*AT1G55130, AT1G55140, AT1G55150, AT1G55152, AT1G55160, AT1G55170, AT1G55175, AT1G55180, AT1G55190, AT1G55200*
VANDAL16	Chr3:2718661-2718663	Chr3-2718662	*TRANS*	5.01E-09	1.72E-03	1.76E-06	0.336	*AT3G08890, AT3G08900, AT3G08910, AT3G08920, AT3G08930, AT3G08940, AT3G08943, AT3G08947, AT3G08950*

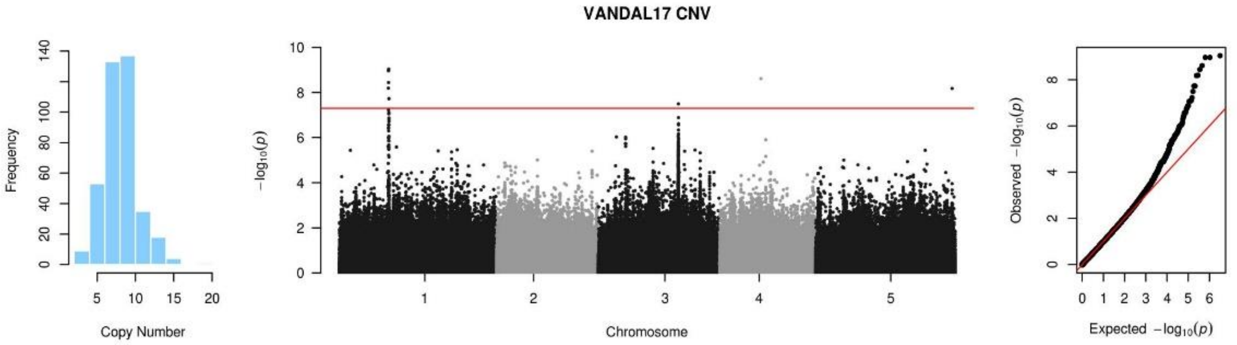

TE	GWAS associated interval	Leading SNP	Type of association	*P-value* Joined	Worldwide	Sweden	MAF	Candidate gene(s)
VANDAL17	Chr1:9512264-9629285	Chr1-9574348	*TRANS*	9.11E-10	2.04E-05	6.71E-06	0.097	.
VANDAL17	Chr4:8006212-8006214	Chr4-8006213	*TRANS*	2.45E-09	1.21E-08	1.51E-02	0.072	*AT4G13780, AT4G13790, AT4G13800, AT4G13810, AT4G13820, AT4G13830, AT4G13840*
VANDAL17	Chr5:26385626-26385628	Chr5-26385627	*TRANS*	6.63E-09	1.85E-14	5.83E-01	0.056	*AT5G65930, AT5G65940, AT5G65950, AT5G65960, AT5G65970, AT5G65980, AT5G65990*

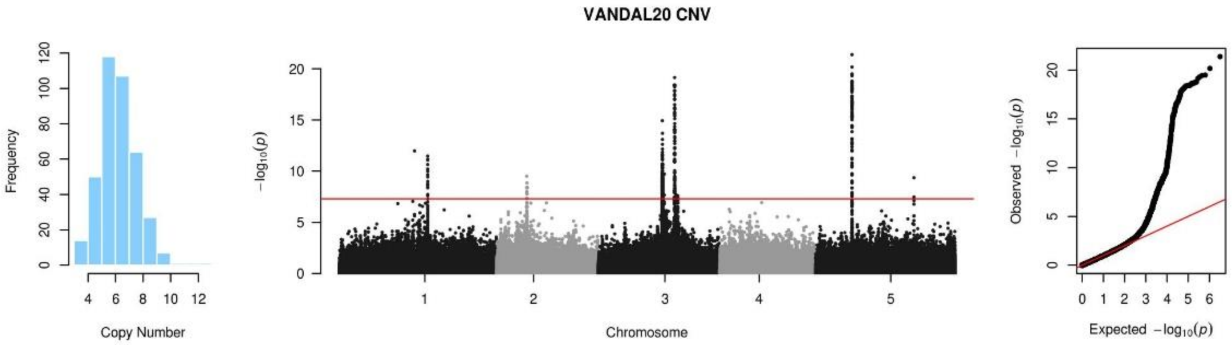

TE	GWAS associated interval	Leading SNP	Type of association	*P-value*			MAF	Candidate gene(s)
				Joined	Worldwide	Sweden		
VANDAL20	Chr1:14600154-14600156	Chr1-14600155	*TRANS*	1.06E-12	NA	1.77E-07	0.100	
VANDAL20	Chr1:17133749-17146953	Chr1-17135470	*TRANS*	6.90E-12	7.17E-06	3.40E-08	0.351	*AT1G45201, AT1G45207, AT1G45215, AT1G45221, AT1G45223, AT1G45229, AT1G45231, AT1G45233, AT1G45234, AT1G45243*
VANDAL20	Chr2:5860912-5905408	Chr2-5860913	*TRANS*	3.13E-10	2.88E-10	4.28E-02	0.064	*AT2G13950, AT2G13960, AT2G13980, AT2G14000, AT2G14045, AT2G14050, AT2G14060*
VANDAL20	Chr3:12377529-12448562	Chr3-12395623	*TRANS*	1.17E-15	2.18E-08	4.39E-11	0.185	*AT3G30768, AT3G30769, AT3G30770, AT3G30775*
VANDAL20	Chr3:12536059-12536061	Chr3-12536060	*TRANS*	2.98E-08	2.24E-07	2.94E-01	0.218	*AT3G30820*
VANDAL20	Chr3:12747790-12767654	Chr3-12767653	*TRANS*	1.92E-10	NA	8.08E-05	0.069	*AT3G31400, AT3G31401, AT3G31402*
VANDAL20	Chr3:14761600-14806561	Chr3-14763896	*CIS*	7.16E-20	1.54E-10	7.17E-11	0.305	
VANDAL20	Chr3:15070929-15070931	Chr3-15070930	*TRANS*	2.06E-08	NA	2.87E-06	0.059	*AT3G43083*
VANDAL20	Chr3:15371779-15371781	Chr3-15371780	*TRANS*	2.55E-08	7.30E-04	2.19E-06	0.174	*AT3G43440*
VANDAL20	Chr5:7027445-7038846	Chr5-7034340	*CIS*	4.15E-22	1.98E-10	1.57E-15	0.479	
VANDAL20	Chr5:19019479-19019481	Chr5-19019480	*TRANS*	4.43E-10	5.91E-08	1.03E-03	0.333	*AT5G46850, AT5G46860, AT5G46870, AT5G46871, AT5G46873, AT5G46874, AT5G46877*

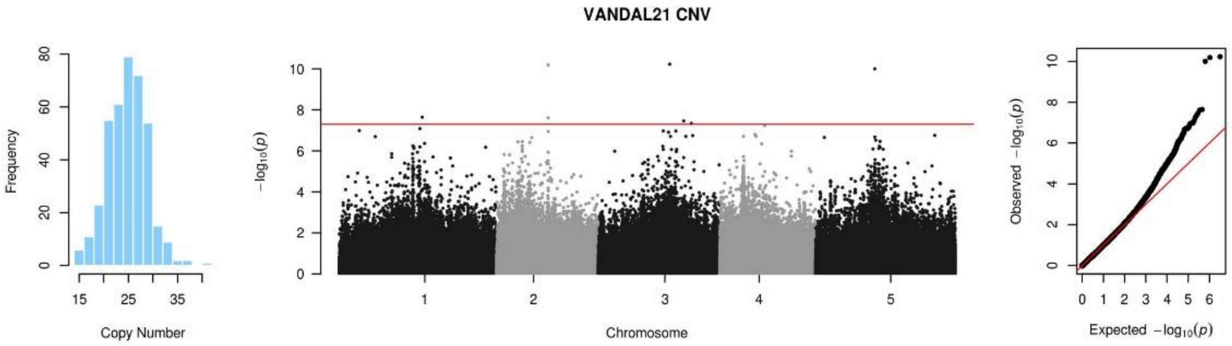

TE	GWAS associated interval	Leading SNP	Type of association	*P-value*			MAF	Candidate gene(s)
				Joined	Worldwide	Sweden		
VANDAL21	Chr1:16073091-16073093	Chr1-16073092	*TRANS*	2.27E-08	2.89E-08	NA	0.228	*AT1G42700, AT1G42710*
VANDAL21	Chr2:10001804-10002742	Chr2-10001805	*CIS*	6.48E-11	2.33E-06	8.92E-06	0.254	
VANDAL21	Chr3:13826287-13826289	Chr3-13826288	*TRANS*	5.90E-11	9.74E-10	9.59E-03	0.228	.
VANDAL21	Chr5:11418711-11418713	Chr5-11418712	*TRANS*	9.97E-11	4.38E-09	2.08E-03	0.349	.
