# Supplementary figures and images for: The Arabidopsis thaliana mobilome and its impact at the species level
Source: eLife. 2016 Jun 3;5:e15716. doi: 10.7554/eLife.15716 (PMC4917339; doi:10.7554/eLife.15716)

# Supplementary file 2. DNA sequence motifs at insertions sites

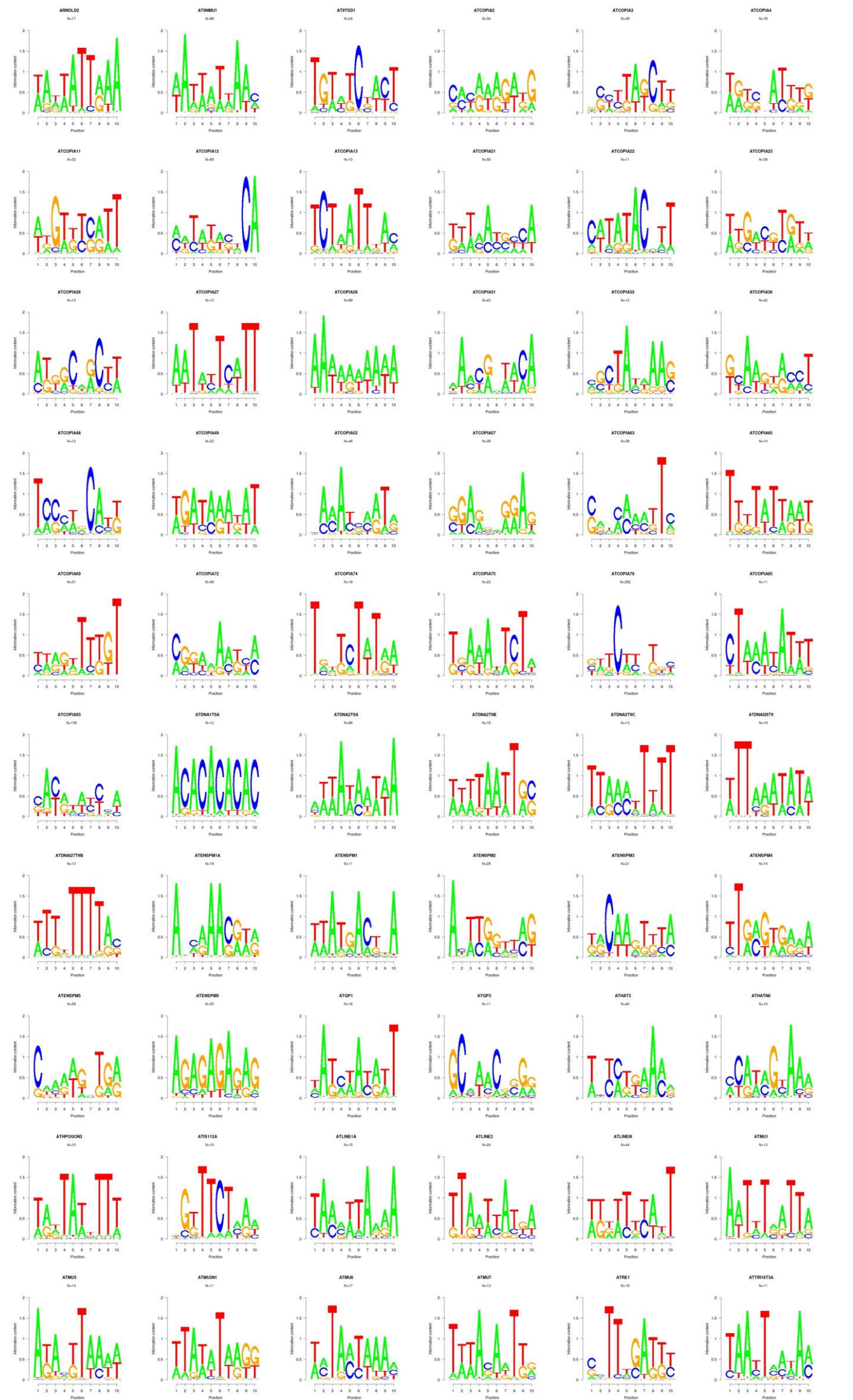

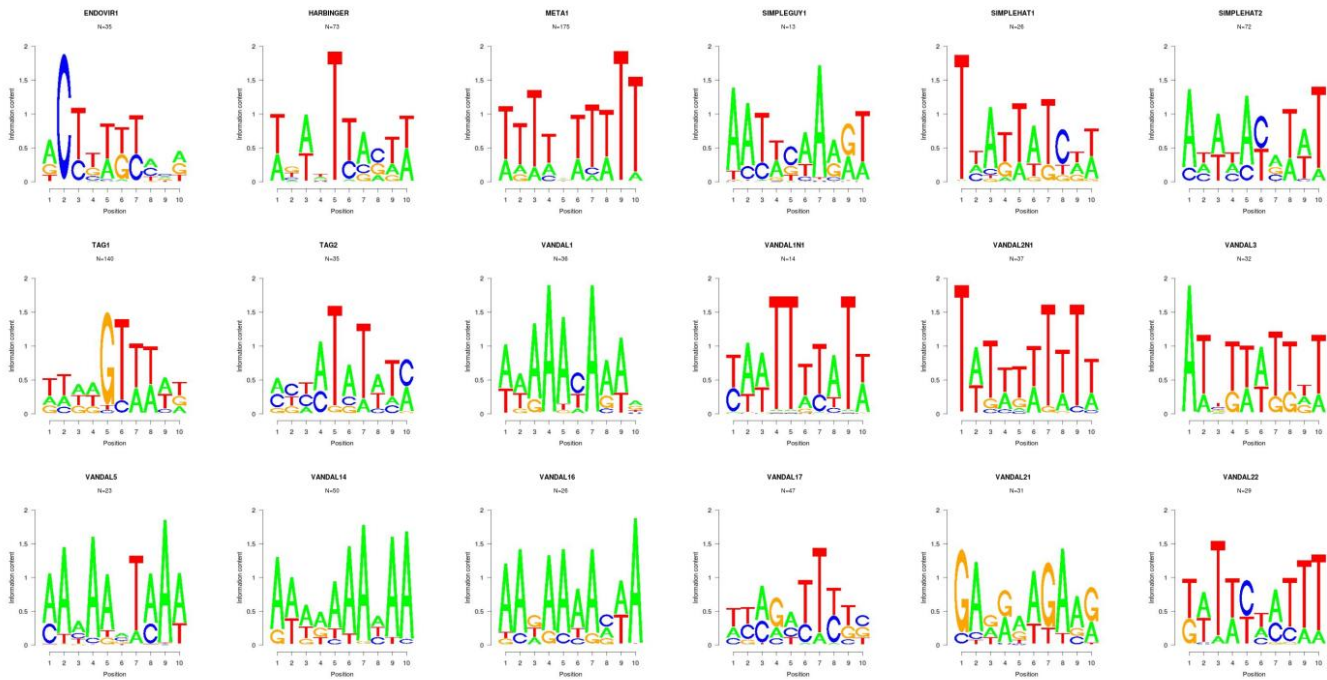

Supplement: Supplementary file 2. — Sequence logo of the overrepresented DNA sequence motifs at insertion sites ( ± 30bp) is shown for the 79 mobile TE families with at least 10 non-reference TE insertions with TSDs. The number of sequences used in each case is indicated. DOI: http://dx.doi.org/10.7554/eLife.15716.024 [file elife-15716-supp2.zip › Supplementary_file2.pdf]
